# Impact of Fiber Orientation in Electrospun PLA Scaffolds on Fluid Dynamics in a Custom Microfluidic Device

**DOI:** 10.1002/adhm.202500378

**Published:** 2025-06-17

**Authors:** Elisa Capuana, Maria Testa, Chiara Di Marco, Francesco Lopresti, Vincenzo La Carrubba

**Affiliations:** ^1^ Department of Engineering University of Palermo Palermo 90128 Italy; ^2^ Department of Biomedicina, Neuroscienze e Diagnostica avanzata (Bind) University of Palermo Palermo 90127 Italy

**Keywords:** computational fluid dynamics (CFD), electrospinning, fiber alignment, liquid permeability, microfluidic devices, Organ‐on‐Chip

## Abstract

Organ‐on‐Chip (OoC) systems are evolving as vital tools in biomedical research, proposing advanced platforms to replicate human tissue microenvironments for drug testing and disease modeling. This study examines how the orientation of polylactic acid (PLA) fibers influences fluid movement in a custom OoC setup. PLA scaffolds are fabricated via electrospinning with either random or aligned fiber orientations. Scanning electron microscopy (SEM) reveals that random scaffolds are 70 µm thick with fibers measuring 1.12 µm, while aligned scaffolds are thinner at 35 µm with fibers of 1.02 µm. Porosity and matrix structure are analyzed to understand the impact of fiber arrangement. Liquid water permeability is tested using a custom three dimensional (3D)‐printed device conforming to ISO 7198:2016 standards. Computational fluid dynamics (CFD) simulations, employing the Porous Media Flow Module and Brinkman's equations, predict flow behavior based on scaffold morphology. A dual‐chamber microfluidic chip integrated with pressure sensors allows real‐time measurements to validate the simulations. Results demonstrate that fiber alignment significantly alters scaffold permeability and flow dynamics. These insights are valuable for tissue engineering, offering a validated framework to design microfluidic devices with tailored fluidic environments optimized for specific scaffold architectures.

## Graphical abstract

Fiber orientation in electrospun scaffolds affects permeability and fluid dynamics in microfluidic devices. Combining experimental data with computational fluid dynamic simulations, we show that aligned fibers enhance flow uniformity, reduce pressure and shear stress variability. These findings provide key insights for optimizing scaffold‐integrated Organ‐on‐Chip platforms, where controlled microenvironments are essential for dynamic cell cultures.

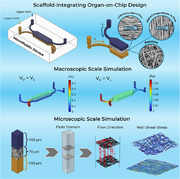

## 1 Introduction

The new Organ‐on‐Chip (OoC) approach replicates vital tissue microenvironments directly within laboratory tests, offering precise control over biomechanical and biochemical stimuli. This innovation facilitates the development of physiologically relevant models for drug testing, disease modeling, and regenerative medicine.^[^ ^1^, ^2^, ^3^, ^4^ ^]^ A key advantage of OoC platforms is their ability to integrate dynamic fluid flow, spatial gradients, and mechanical cues, providing a more accurate representation of native tissues compared to traditional static cultures. Current out‐of‐chamber systems often rely on basic two‐dimensional (2D) monolayer cultures, which lack the complexity of real extracellular matrix (ECM) structures essential for native tissue functions.^[^ ^5^ ^]^

Research demonstrates that connecting three‐dimensional (3D) scaffold materials to microfluidic platforms enhances cellular adhesion and promotes natural development.^[^ ^6^, ^7^ ^]^ These synthetic matrices mimic the physical structure and bioactive signals of living tissues, creating an environment conducive to cell growth. Among fabrication techniques, electrospinning stands out for its ability to produce scaffold structures with micro‐ and nanoscale fibers that replicate ECM properties.^[^ ^8^, ^9^ ^]^ By adjusting polymer dosage, electrical current settings, and fluid pumping speeds, electrospinning allows precise control over fiber dimensions, pore size, and material toughness.^[^ ^10^, ^11^, ^12^ ^]^ Studies confirm that electrospun scaffolds are highly effective in tissue engineering due to their versatility in structuring cells and guiding tissue growth.^[^ ^13^ ^]^

Fiber alignment within scaffolds is a critical factor for microfluidic applications. The orientation of fibers in electrospun scaffolds significantly influences cellular interactions and material behavior.^[^ ^14^, ^15^ ^]^ Aligned fiber scaffolds guide cell orientation and elongation, making them particularly advantageous for engineering anisotropic tissues such as skeletal muscle, tendons, ligaments, and nerve tissues. These scaffolds can also enhance unidirectional fluid transport, which may be beneficial for perfusion‐based tissue models.^[^ ^16^, ^17^ ^]^ In contrast, random fiber scaffolds exhibit an isotropic organization that promotes uniform cell distribution and facilitates omnidirectional diffusion of nutrients and metabolites—beneficial for applications such as wound healing and soft tissue engineering.^[^ ^18^, ^19^ ^]^

Beyond biological considerations, water permeability is a key parameter influencing scaffold functionality within microfluidic environments. In OoC models, scaffold permeability directly affects fluid dynamics, shear stress distribution, and mass transport efficiency—all critical for sustaining cell viability and function.^[^ ^20^ ^]^ Cellular dysfunction can occur when nutrient movement within scaffolds is restricted, leading to oxygen deprivation or the accumulation of harmful substances. Conversely, excessive scaffold permeability can compromise both structural integrity and protective properties. Despite its significance, the impact of fiber alignment on scaffold permeability remains underexplored in the field of tissue engineering. Optimizing microfluidic devices and analyzing them through computational fluid dynamics (CFD) simulations help bridge this knowledge gap. CFD models provide a detailed understanding of how scaffold materials influence fluid movement patterns in microfluidic systems, offering precise reports on flow velocity, pressure zones, and force levels.^[^ ^21^, ^22^, ^23^, ^24^ ^]^ By integrating experimental permeability measurements with CFD simulations, researchers can establish quantitative relationships between scaffold architecture and fluid transport characteristics. This technique significantly improves the design of scaffolds to support tissue engineering processes within microfluidic systems.

In this study, we investigate how fiber direction affects fluid flow through polylactic acid (PLA) scaffold structures used in microfluidic systems. The research is structured into two main phases. First, we experimentally quantify scaffold permeability by fabricating electrospun scaffolds with controlled fiber alignments—specifically aligned versus random configurations—using optimized processing parameters. A custom‐designed microfluidic setup is developed to accurately measure the water permeability of these scaffolds under controlled flow conditions. The collected permeability data are analyzed to determine the influence of fiber orientation on mass transport properties. In the second phase, CFD simulations are employed to integrate the experimentally determined permeability values into models that simulate fluid flow within a dual‐flow microfluidic device. Key hydrodynamic parameters—including velocity distribution, pressure drop, and shear stress at the scaffold interface—are analyzed to assess the impact of fiber alignment on overall microfluidic device performance. This approach enables the identification of design guidelines for scaffold‐based organ‐on‐chip systems. By combining experimental characterization with numerical modeling, this study aims to elucidate the relationship between fiber orientation, scaffold permeability, and microfluidic flow dynamics—ultimately contributing to optimizing scaffold‐integrated organ‐on‐chip platforms for tissue engineering applications.

## 2 Results

### 2.1 Scaffold Characterization: Porosity, Scanning electron microscopy (SEM), and Permeability

**Figure 1:**
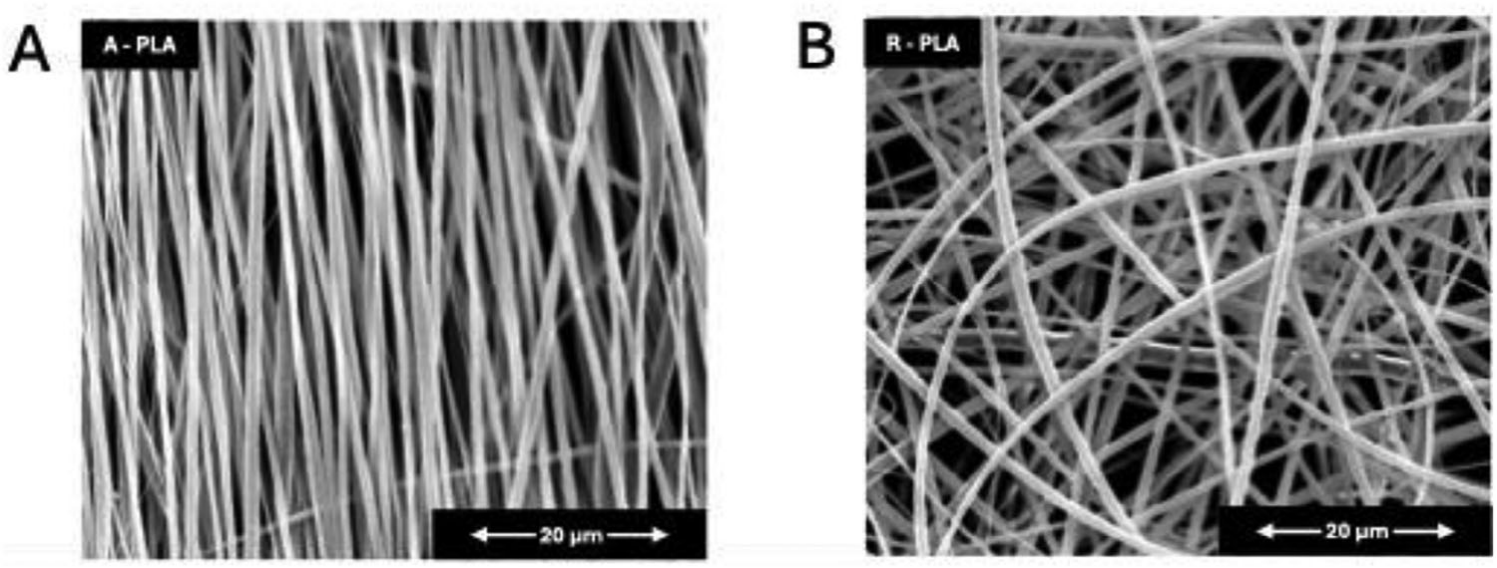
SEM images of electrospun PLA scaffold with: A) fibers with aligned orientation (A‐PLA, left); and B) fibers with randomized orientation (R‐PLA, right). Scale bars: 20 µm.

SEM was employed to analyze the morphology of PLA scaffolds, focusing on their alignment and fiber characteristics. The study compared aligned (A‐PLA) and random (R‐PLA) fiber orientations, as shown in **Figure** **1**A,B, respectively. The fiber diameter size distribution plot and the distribution of fiber orientation were further detailed in Figures S1A,B (Supporting Information).

Both aligned and random scaffolds exhibited a homogeneous cylindrical fiber shape, without the presence of beads or other morphological defects. The fiber diameter distribution plot (Figure S1A, Supporting Information) highlighted that the aligned fibers had a slightly narrower distribution of the fiber diameter compared to the random ones, although both the systems present a peak of frequency ≈1 µm. Specifically, the average fiber diameter for random scaffolds was 1.12 µm, while for aligned scaffolds, it was 1.02 µm.^[^ ^25^ ^]^ Fiber alignment was achieved using a rotating drum collector operating at 3000 rpm. In such a method, mechanical forces are used to draw the fiber as it is deposited on the spinning drum thereby inducing fiber alignment.^[^ ^26^ ^]^ This method was qualitatively and quantitatively assessed, with the alignment efficacy visible in Figure 1A,B, and the orientation distribution (Figure S1B, Supporting Information) showing a narrow peak ≈0° for aligned fibers.

The porosity (φ)of the PLA scaffolds, both in random and aligned configurations, was evaluated using established methods in scientific literature.^[^ ^27^ ^]^ The results indicated that the porosity of the random scaffolds was 75%, while that of the aligned scaffolds was 64% (**Table** **1** **)**. These differences in porosity for aligned and randomly oriented fibers are consistent with the results of other scholars for similar systems and it can be ascribed to the denser fiber network formed by aligned fibers (Figure 1A) compared to randomly oriented ones (Figure 1B).^[^ ^28^ ^]^

**Table 1:** Permeability (K) and porosity (φ) values calculated for scaffolds with aligned and random fibers expressed as mean ± standard deviation (SD) (N = 5).

	Aligned fibers	Random fibers
K × 10^−15^ [m^2^]	50.6 ± 11.6	4.69 ± 2.35
φ [%]	75.5 ± 2	63.7 ± 3

Permeability (K) measurement results, obtained following the ISO 7198:2016 standard, were derived from tests conducted on porous scaffolds, distinguishing between aligned and random orientations. For aligned scaffolds, the average permeability was 5.06 × 10^−14^ m^2^, with a standard deviation of 1.16 × 10^−14^ m^2^. In contrast, the random fiber scaffolds exhibited an average permeability was 4.69 × 10^−15^ m^2^ and a standard deviation of 2.35 × 10^−15^ m^2^. Data analysis indicated that random scaffolds possess an order of magnitude higher permeability than aligned ones. Table 1 summarizes these permeability results alongside the porosity values obtained for both types of scaffolds.

### 2.2 OoC Design

**Figure 2:**
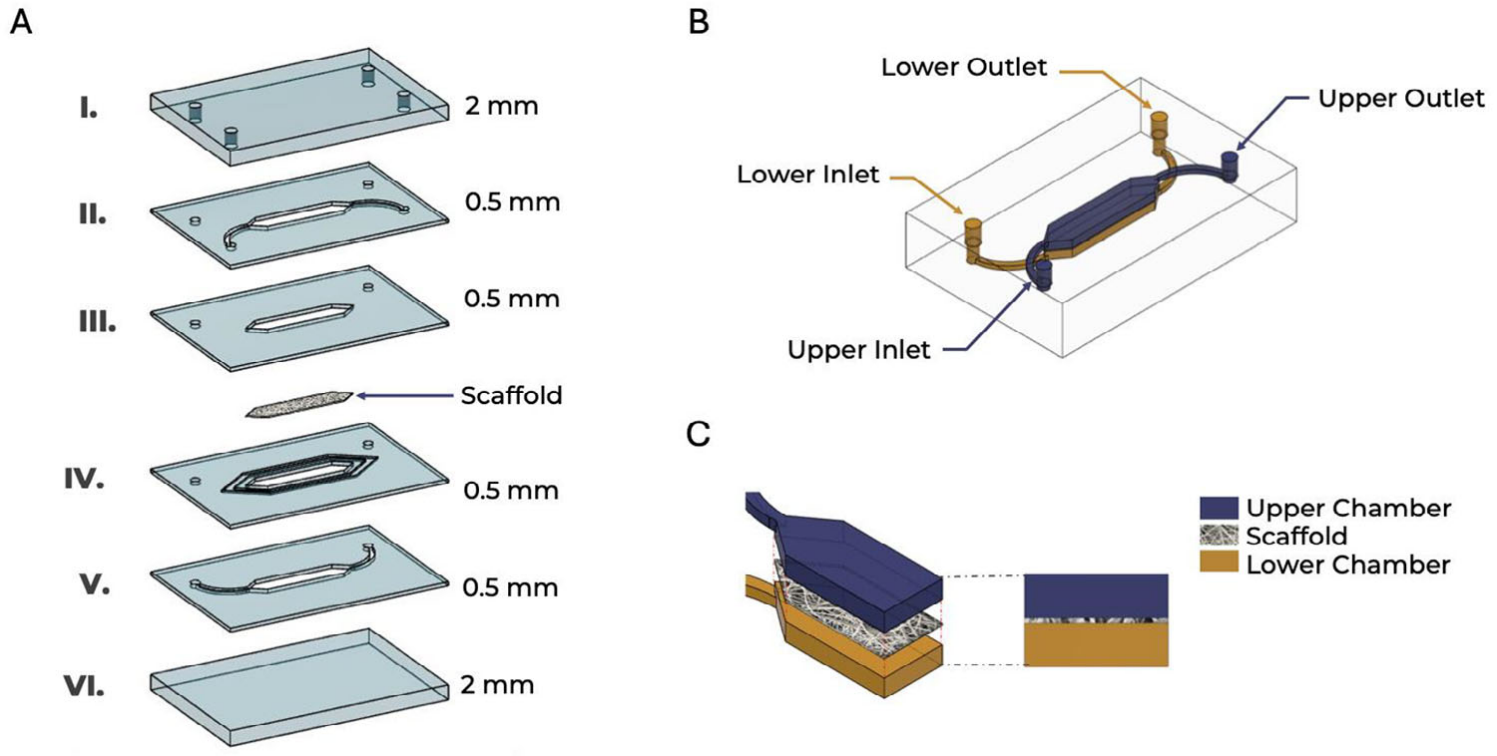
Design and conceptualization of the microfluidic device: A) Exploded view of the microfluidic device showing the seven stacked layers with varying thicknesses (0.5 and 2 mm); B) the assembled device includes dedicated inlet and outlet ports for both microfluidic chambers; C) Cross‐sectional view of the device highlighting the spatial organization of the upper and lower chambers, with the scaffold positioned as a barrier between compartments.

Seven layers with varying thicknesses (0.5 and 2 mm) (**Figure** **2**A) were CAD‐designed to form a dual‐chamber system in which the separation between the upper and lower compartments is ensured by an electrospun scaffold integrated within the structure (Figure 2B,C). Each layer serves a specific function within the device architecture: the first upper layer provides mechanical stability, closure, and holes for inlet and outlet connection with the pump (Figure 2A). The second and third layers integrate the inlet and outlet channels of the upper chamber and the upper chamber itself (blue labeled in Figure 2B). The fourth layer houses the electrospun scaffold, which acts as a barrier between the two compartments. The fifth, sixth, and seventh layers comprise the inlet and outlet channels of the lower chamber and form the structural base of the device (yellow labeled in Figure 2B). The cross‐sectional view in Figure 2C illustrates the detailed organization of the chambers and the position of the scaffold, highlighting the compartmentalization strategy adopted to maintain distinct environments within the same device.

A detailed technical drawing with all dimensional specifications is provided in Figure S2 (Supporting Information).

### 2.3 Computational Simulation

#### 2.3.1 CFD Simulations at Macroscopic Scale

The results of the macroscopic scale simulations demonstrate variations in velocity and pressure based on fiber arrangement (random vs aligned) and flow conditions (either equal velocities or a tenfold increase in one chamber). The following figures focus on the results of the R‐PLA scaffold, as those for A‐PLA ones exhibit ed similar characteristics. The small differences observed between the two morphologies are discussed below for each flow configuration.

**Figure 3:**
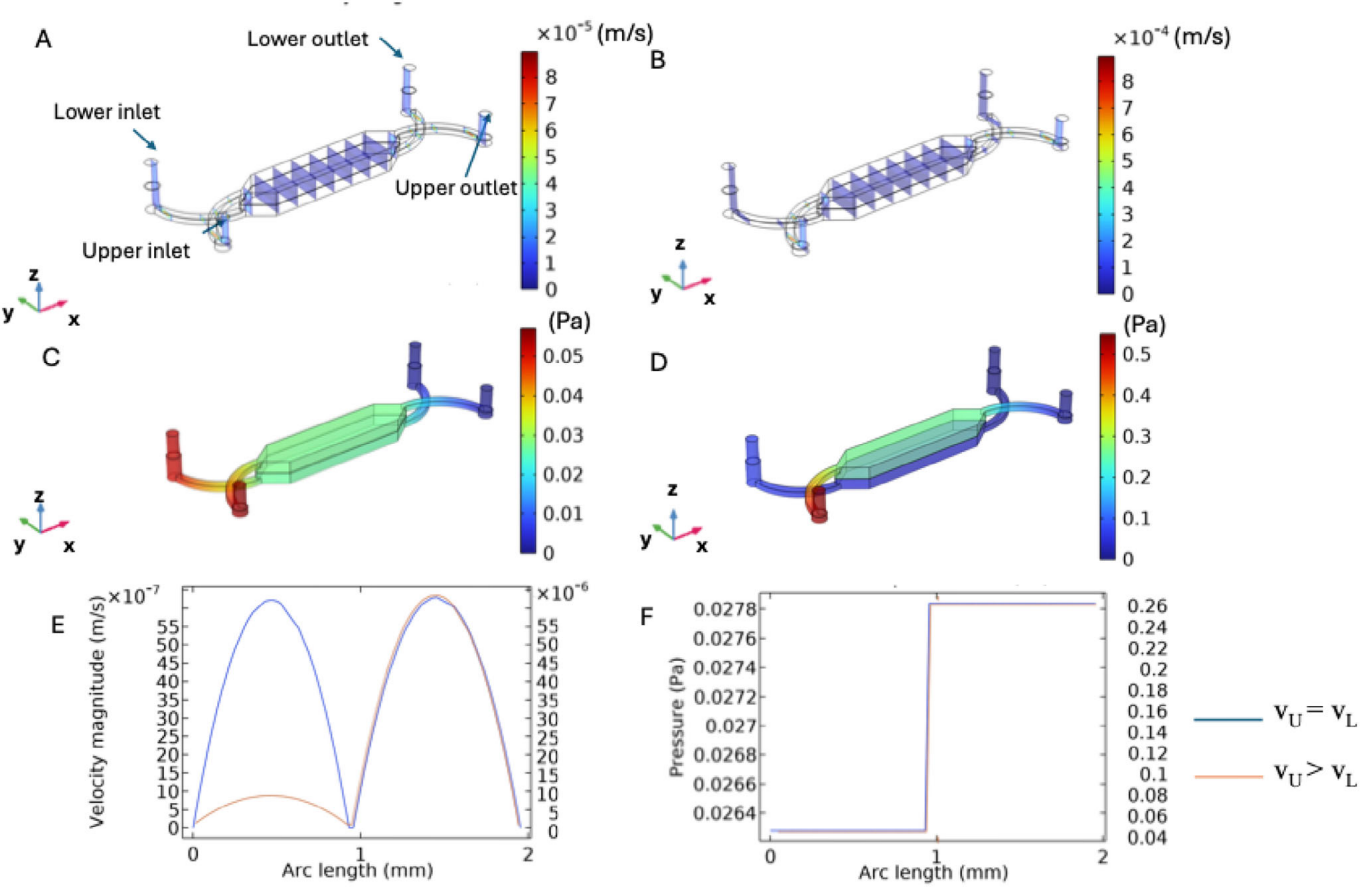
Simulation results at the macroscopic scale for random and aligned scaffolds when v_U_ = v_L_ (corresponding to a flow rate of ≈1 mL day^−1^) and v_U_ > v_L_ (corresponding to a flow rate of ≈10 and 1 mL day^−1^): A) velocity along five yz planes when v_U_ = v_L_; B) velocity along five yz planes when v_U_ > v_L_; C) pressure distribution when v_U_ = v_L_, D) pressure distribution when v_U_ > v_L_; E) velocity profile along z for both flow configurations (blue curve refers to v_U_ = v_L_, orange curve refers to v_U_ > v_L_ applied along the secondary y‐axis); F) pressure profile along z for both flow configurations (blue curve refers to v_U_ = v_L_, orange curve refers to v_U_ > v_L_ applied along the secondary y‐axis). v_U_ and v_L_ indicate the flow velocity in the upper and lower chambers, respectively. Arc length indicates the distance along the z‐axis of the device, measured in millimeters, where 0 mm represents the bottom and 2 mm the top, and along which the velocity and pressure profiles were measured.

In **Figure** **3**A, when *the velocity in the upper chamber (* *v_U_)= the velocity in the lower chamber (v_L_* *)*, the velocity along yz planes shows a relatively uniform distribution with minimal variations near the scaffold walls, indicating stable and uniform flow propagation. Conversely, when *v_U_ > v_L_* (Figure 3B), a significant velocity gradient emerges, with higher velocities near the upper scaffold region, leading to accelerated flow in that area. In Figure 3C (*v_U_ = v_L_*), the pressure is distributed along the scaffold with a slight gradient, gradually decreasing from the inlet to the outlet zone. However, when *v_U_ > v_L_*, the pressure distribution (Figure 3D) a steeper pressure gradient is observed due to increased upper‐chamber velocity, resulting in a more pronounced pressure drop across the scaffold. The velocity profile along the z‐axis (Figure 3E) displays a symmetrical sinusoidal pattern when *v_U_ = v_L_* (blue line), indicative of balanced flow with minimal pressure differences. In contrast, when *v_U_ > v_L_* (orange line), substantial asymmetry is noted, with pronounced peaks near the top scaffold surface, suggesting localized turbulence. In Figure 3F, the pressure profile along z (i.e., the transmembrane pressure) is nearly linear for both configurations but tilts sharply when *v_U_ > v_L_* (orange line), reflecting how increased upper velocity affects overall flow dynamics.

**Figure 4:**
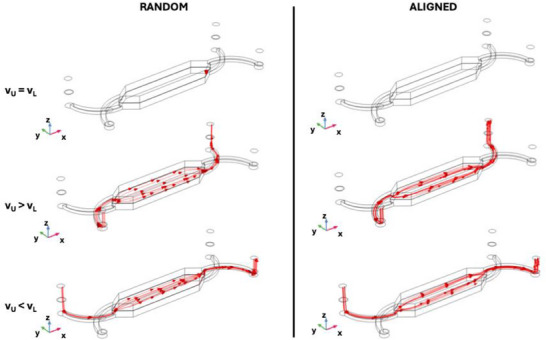
Streamlines across the scaffold obtained from simulations at the macroscopic scale for random and aligned scaffolds integrated into the double‐chamber OoC. Three flow configurations concerning different velocity values in the upper (v_U_) and lower (v_L_) chambers were analyzed: v_U_ = v_L_, v_U_ = 10 × v_L_, and v_L_ = 10 × v_U_. Distinct flow profiles were observed for the two fiber arrangements when the velocities in the upper and lower chambers differed.

**Figure** **4** illustrates the streamlines of the liquid flow passing through the scaffold. When the flow rates above and below the scaffold are equal (*v_U_ = v_L_*), minimal liquid transfer occurs between chambers. This finding indicates the possibility of designing systems that facilitate molecular exchange primarily through diffusion while minimizing convective contributions. Conversely, when the flow rates between the upper and lower chambers differ, streamlines show significant liquid transfer from the two chambers, with aligned fibers creating two preferred flow paths, whereas random fibers exhibited uniform distribution across scaffold width (Figure 4, *v_U_ > v_L_* and *v_U_ < v_L_*)Specifically, when the velocity in the upper chamber is 10 times lower than in the lower chamber, flow passes through the scaffold from the lower to the upper chamber for both fiber configurations. The configuration with the tenfold difference in flow rates between the upper and lower chambers can therefore be employed to model scenarios requiring convective transport. Moreover, using Darcy's law and transmembrane pressure results (ΔP, Figure 3F; Figure S3B, Supporting Information), flow rates across scaffolds were calculated. Findings revealed that when *v_U_ = v_L_*, the flow rate through the R‐PLA scaffold was ≈0.013 µL h^−1^, while for the A‐PLA scaffold, it was 0.13 µL h^−1^. When *v_U_ = 10 × v_L_*, flow rate results were 1.78 µL h^−1^ for R‐PLA and 18.65 µL h^−1^ for A‐PLA. Finally, when *v_L_ = 10 × v_U_*, the flow through the scaffold (from the lower to the upper chamber for this flow configuration) measured 1.62 µL h^−1^ for random fibers and 17.5 µL h^−1^ for aligned fibers. These results, confirming the streamline plots results in terms of flow direction across the scaffold and the permeability measurements, are further illustrated in Figure 10, which compares the computational and experimental flow rates across the scaffold.

#### 2.3.2 CFD simulations at the microscopic scale

**Figure 5:**
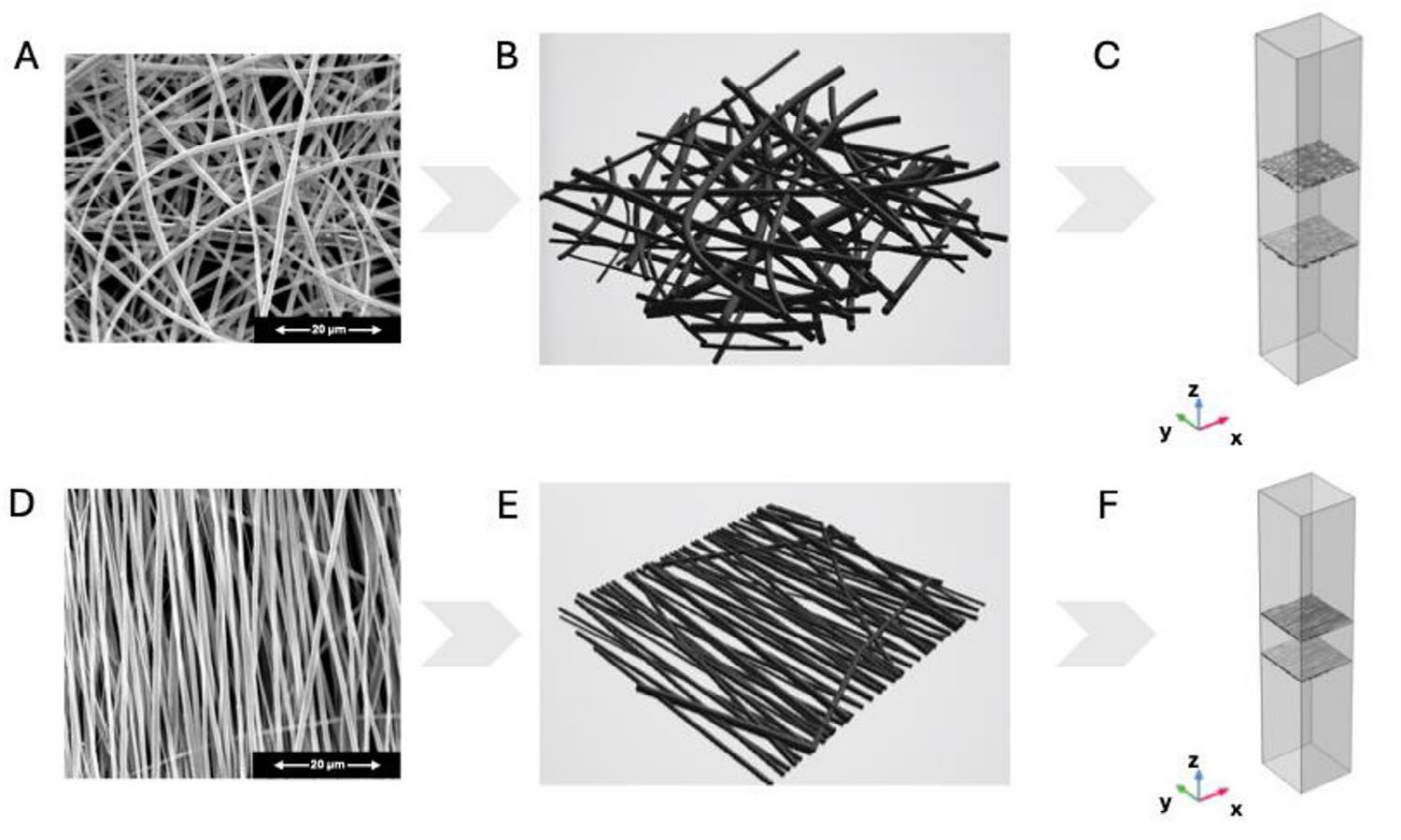
Steps to obtain the geometry for the micro‐scale simulation: A) SEM image of a random PLA scaffold, B) 3D reconstruction of the arrangement of the random fibers, C) coupling of the random fibers with two fluid volumes in the upper and lower regions, along with a porous solid volume between the fibers (total thickness of the scaffold = 70 µm); D) SEM image of an aligned PLA scaffold, E) 3D reconstruction of the arrangement of the aligned fibers, F) coupling of the aligned fibers with two fluid volumes in the upper and lower regions, along with a porous solid volume between the fibers (total thickness of the scaffold = 35 µm).

The steps to obtain the final microscopic configurations of random and aligned fibers are illustrated in **Figure** **5**. Specifically, the scaffold, acting as the porous medium, is positioned between two chambers. The two layers of Figure 5C and Figure 5F correspond to the surfaces where the scaffold directly interfaces with the fluid within these chambers. The lower layer is the surface of the scaffold in direct contact with the fluid in the lower chamber, while the upper layer is the surface in contact with the fluid in the upper chamber. Hence, these layers represent the transition zones where fluid enters or exits the porous scaffold matrix. The bulk material of the scaffold, located between these two interface layers, was modeled as a homogeneous porous medium with measured properties such as porosity and permeability.

For each simulated combination of inlet velocities in the two chambers and fibers arrangement, results are presented in terms of velocity, velocity profile along the z‐axis, pressure, pressure profile along the z‐axis, and shear stress on the top and bottom surfaces of the scaffold fibers in contact with the fluid. Streamlines suggest a passage from lower to upper chambers; specifically, random scaffolds exhibit more dispersed flow with reduced density reflecting decreased velocity in the upper chamber and less intense flow. In contrast, for aligned scaffolds with fibers perpendicular to flow direction, streamlines are more concentrated towards the lower chamber but show reduced density; while for aligned scaffolds with fibers directed as flow, direction remains well directed albeit at lower streamline density.

**Figure 6:**
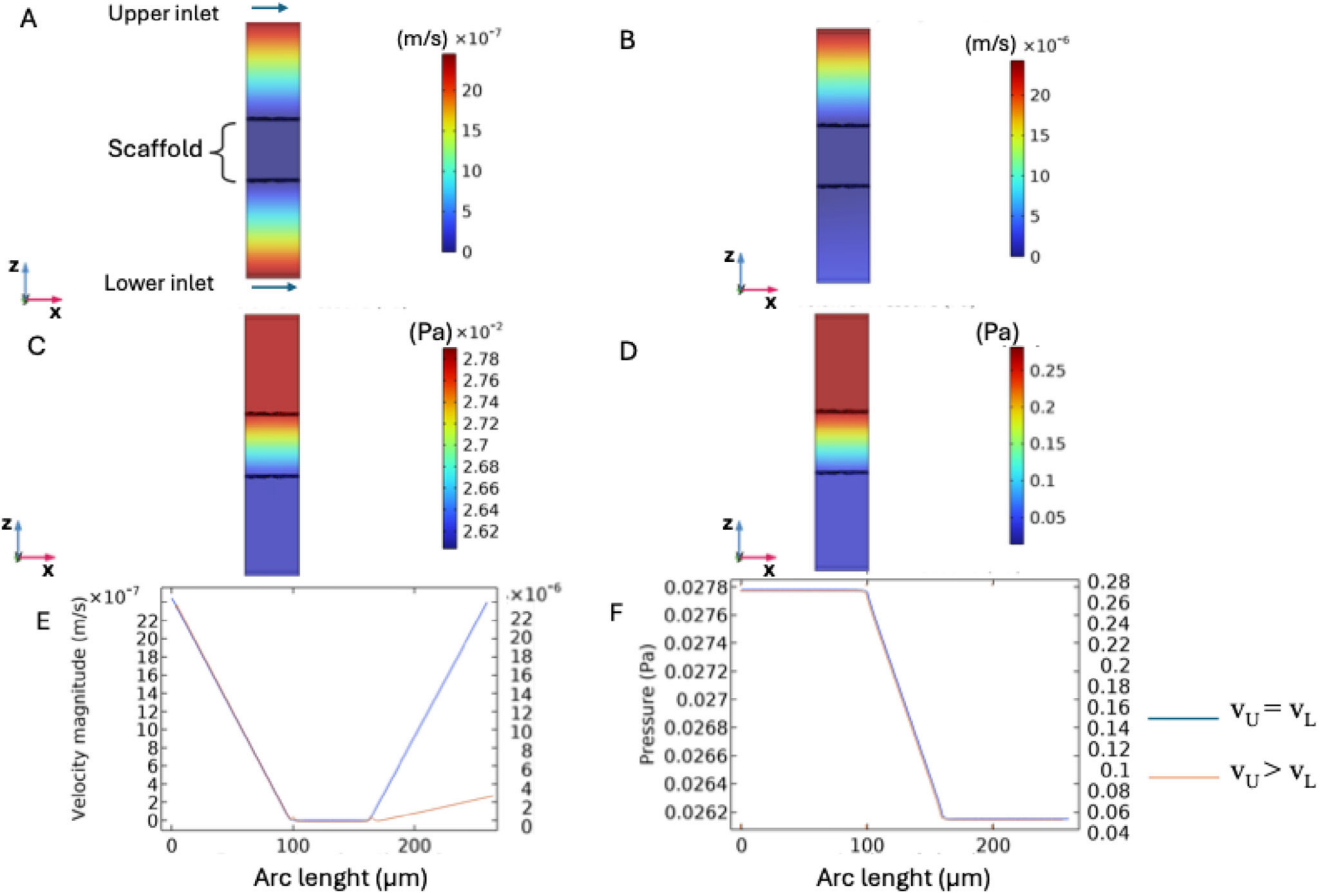
Simulation results at the microscopic scale for random and aligned scaffolds: A) velocity distribution when v_U_ = v_L_; B) velocity distribution when v_U_ > v_L_; C) pressure distribution when v_U_ = v_L_, D) pressure distribution when v_U_ > v_L_; E) velocity profile along z for both flow configurations (blue curve refers to v_U_ = v_L_, orange curve refers to v_U_ > v_L_ applied to the secondary y‐axis); F) pressure profile along z for both flow configurations (blue curve refers to v_U_ = v_L_, orange curve refers to v_U_ > v_L_ applied to the secondary y‐axis). v_U_ and v_L_ indicate the flow velocity in the upper and lower chambers, respectively. Arc length indicates the distance along the z‐axis of the domain, measured in microns, where 0 mm represents the bottom and 270 µm the top, and along which the velocity and pressure profiles were measured.

From the CFD analysis, for all the fiber arrangements (random fibers, aligned fibers parallel to flow, and fibers perpendicular to flow), results were similar across all fiber configurations. Therefore, subsequent figures focus on the randomly orientated scaffold. **Figure** **6**A,C,E (blue line), and Figure 6F (blue line) present micro‐scale simulation results for a random scaffold under specified velocity conditions (*v_U_ = v_L_*). The velocity distribution (Figure 6A) shows an initial decrease followed by an increase, as depicted in the velocity magnitude profile along the arc (Figure 6E, blue line). The pressure distribution (Figure 6C) shows a significant gradient along the z‐direction, decreasing linearly until stabilization (Figure 6F, blue line). Concurrently, these results indicate a complex flow behavior within the scaffold influenced by variations in pressure and velocity along its length, suggesting that scaffold configuration directly affects transport.

Figure 6B,D,E (orange line, secondary y‐axis), and Figure 6F (orange line, secondary y‐axis) present results from microscale simulations for random and aligned scaffolds when v_U_ = 10 × v_L_. Velocity (Figure 6B,E, orange line) increases dramatically near the upper surface, indicating highly accelerated flow, while the pressure profile (Figure 6D,F, orange line) shows a significant decrease along the z‐axis with a pronounced gradient. This suggests flow behavior is strongly influenced by velocity misalignment between the upper and lower chambers.

**Figure 7:**
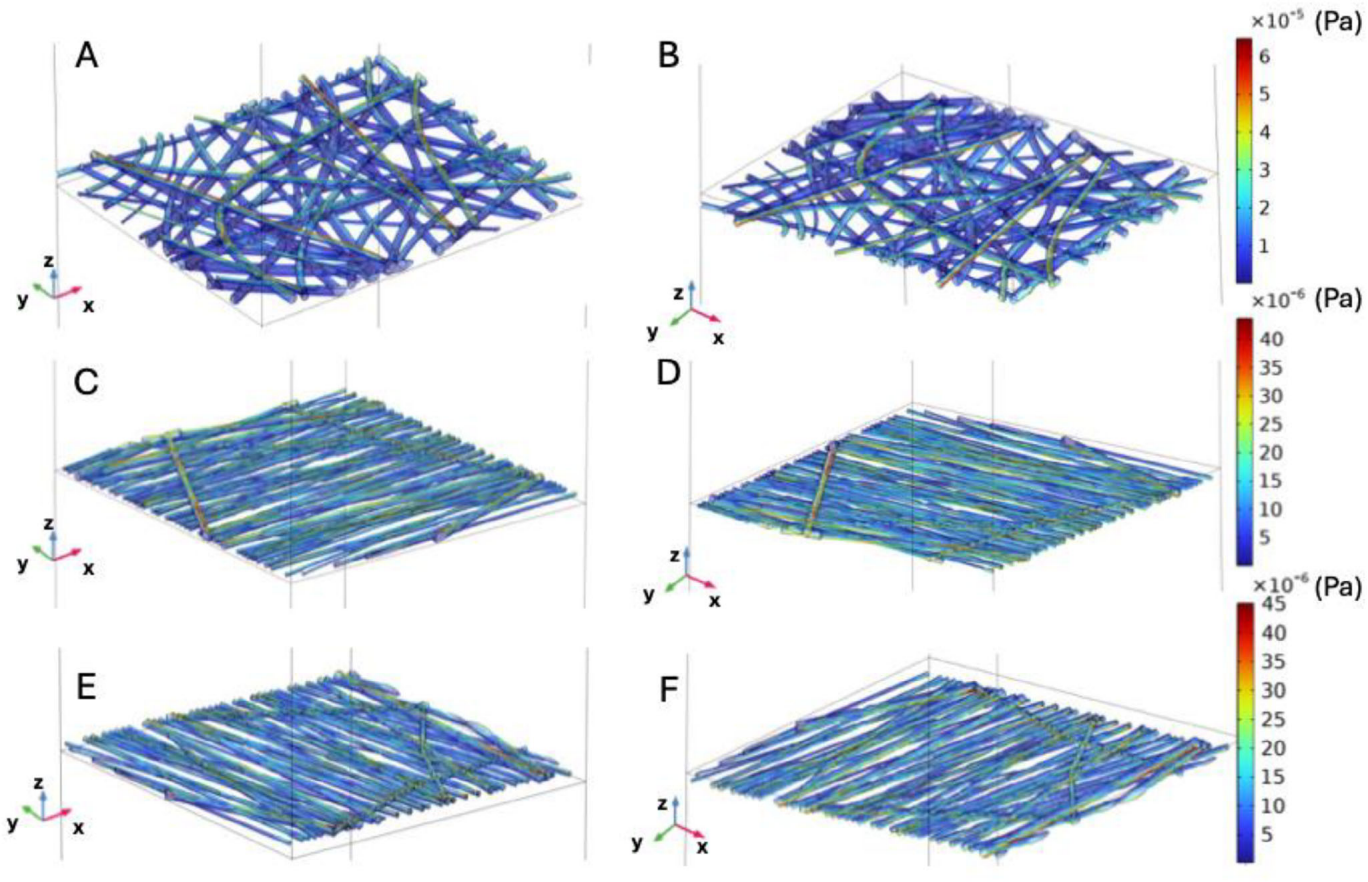
Shear stress on the fibers when v_U_ = v_L_: A) upper surface of the random scaffold, B) lower surface of the random scaffold, C) upper surface of the aligned scaffold with fibers aligned to the direction of flow, D) lower surface of the aligned scaffold with fibers aligned to the direction of flow, E), upper surface of the aligned scaffold with fibers perpendicular to the direction of flow, F) lower surface of the aligned scaffold with fibers aligned to the direction of flow.

When analyzing shear stress along the fibers, under the *v_U_ = v_L_* condition, greater shear stress was observed in random scaffolds, followed by those aligned perpendicular to the flow direction, and finally, those aligned in the flow direction (**Figure** **7**). Moreover, in the random scaffold (Figure 7A,B), the shear stress is unevenly distributed with higher intensity concentrated in specific areas at both the top (Figure 7A) and bottom surfaces (Figure 7B). In contrast, in scaffolds with fibers aligned parallel to the flow direction, there is a slight difference in stress intensity between the top and bottom surfaces, with a greater concentration on the top surface. For scaffolds with fibers aligned perpendicular to the flow direction (Figure 7E,F), shear stress predominantly aligns along the flow direction, with a more consistent distribution than in random scaffolds. The upper surface (Figure 7E) shows a stress distribution similar to that of the lower surface (Figure 7F) but with slightly reduced intensity. This perpendicular alignment promotes stress redistribution along the main axis of flow, reducing local variations compared to R‐PLA scaffolds.

**Figure 8:**
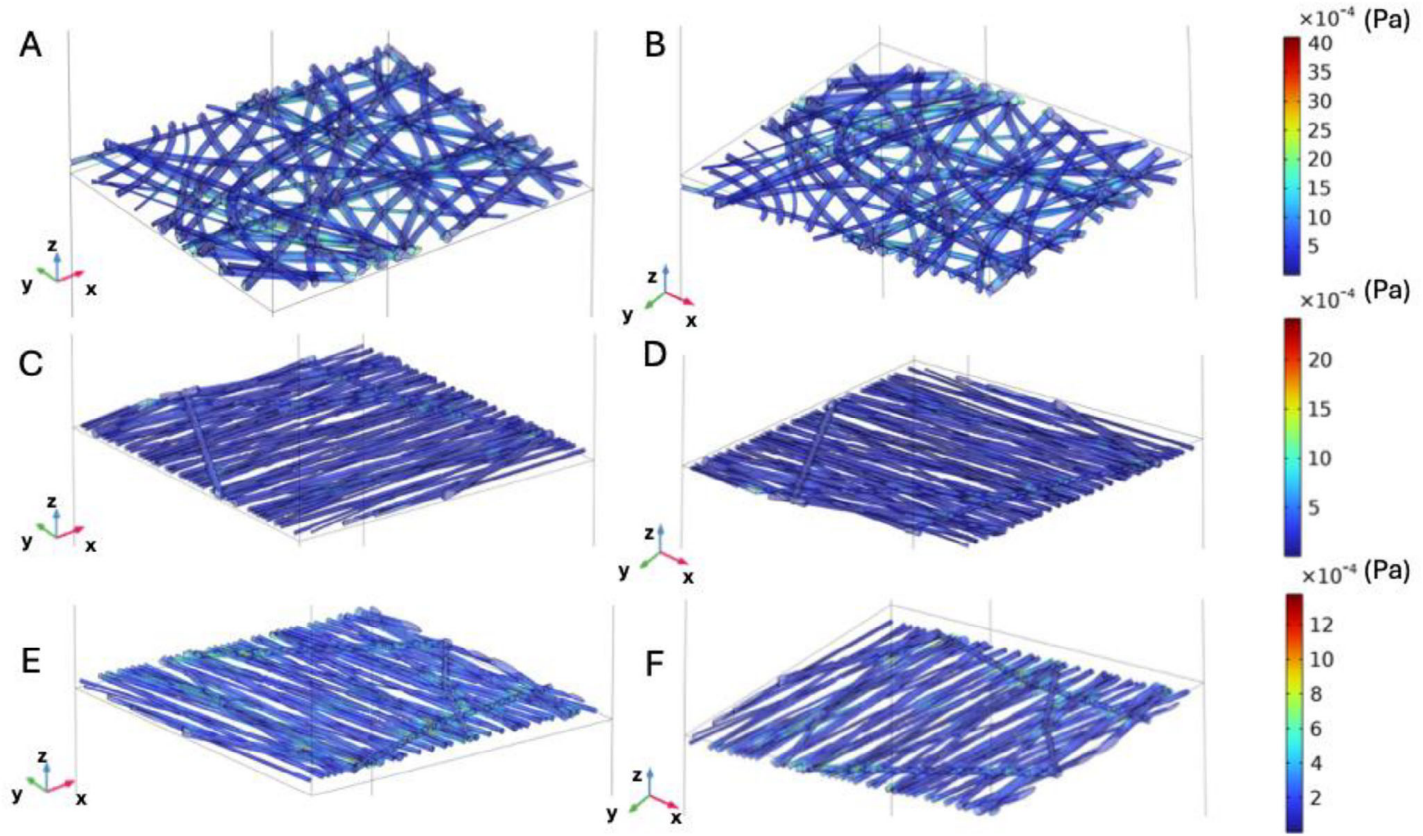
Shear stress on the fibers when v_U_ = 10 × v_L_: A) upper surface of the random scaffold, B) lower surface of the random scaffold, C) upper surface of the aligned scaffold with fibers aligned to the direction of flow, D) lower surface of the aligned scaffold with fibers aligned to the direction of flow, E), upper surface of the aligned scaffold with fibers perpendicular to the direction of flow, F) lower surface of the aligned scaffold with fibers aligned to the direction of flow.

**Figure** **8** illustrates the shear stress distribution on fibers when *v_U_ = 10 × v_L_*. The random scaffold (Figure 8A,B) exhibits high shear stress, particularly at the top surface, indicating intense and inhomogeneous fluid‐scaffold interaction. The scaffold aligned parallel to flow (Figure 8C,D) shows intermediate shear stress levels with a more uniform distribution than random scaffolds but still features concentrated stress points along the flow direction. Finally, scaffolds aligned perpendicular to flow (Figure 8E,F) show lower shear stress with a more uniform distribution reflecting less concentrated interaction between fluid and fibers.

When *v_L_ = 10 × v_U_* (Figure S4, Supporting Information), velocity and pressure profiles along the z‐direction were similar across all fiber configurations. Greater shear stress was observed in scaffolds with fiber aligned in the flow direction followed by those perpendicular to it; finally, random scaffolds exhibited the lowest shear stress (Figure S5, Supporting Information).

**Figure 9:**
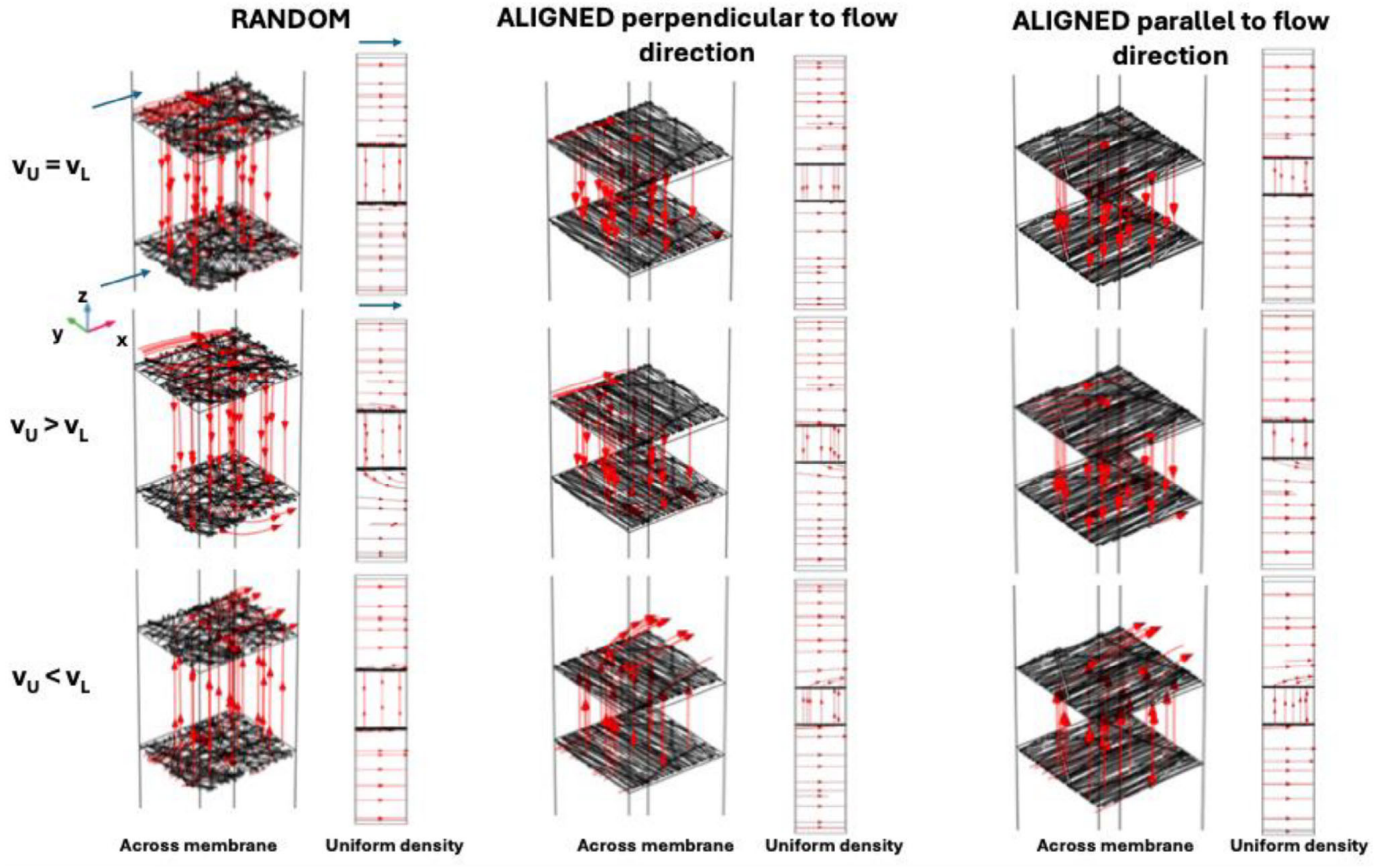
Streamline (red lines) plots obtained from simulations at the microscopic scale for random and aligned scaffolds. For each configuration, two plots were examined: one considering streamlines across the membranes and another with uniform density, indicating the direction and relative intensity of fluid flow. For the aligned scaffold, both flow configurations—fibers aligned with the flow direction and perpendicular to it—were considered. The three flow configurations analyzed involved different values of velocities in the upper (v_U_) and lower (v_L_) chambers. The blue arrows in the first figure (top‐left) represent the inlet direction of the flow for both streamline plots and can be extended to all other images.

Streamlines were also analyzed for both random and aligned scaffolds under different flow and velocity configurations in the upper (*v_U_*) and lower (*v_L_*) chambers. Specifically, conditions for *v_U_ = v_L_, v_U_ > v_L_*, and *v_U_ < v_L_* were examined, with the A‐PLA scaffolds with fibers aligned both perpendicularly and parallel to the flow direction. Each configuration is presented across the scaffold and with uniform streamline density (**Figure** **9**). As shown in Figure 9, random scaffolds with *v_L_ = v_U_* demonstrate relatively uniform flow across the scaffold with a constant distribution, indicating balanced flow without significant directional preferences. For scaffolds aligned perpendicular to flow, the flow direction is maintained but exhibits slight tortuosity. In contrast, scaffolds aligned parallel to the flow show highly directed flow along the fiber directions, with parallel streamlines indicating efficient and minimally dispersed fluid transport. When *v_U_ > v_L_*, streamlines for random scaffolds indicate concentrated downward‐directed flow with increased density, signaling rapid fluid transport from upper to lower chambers. For scaffolds with fibers aligned perpendicular to flow direction, flow is highly directed and concentrated with significantly increased density indicative of fluid acceleration favored by perpendicular fiber alignment. For scaffolds with fibers aligned in the direction of flow, the flow remains maximally concentrated along the direction of the fibers with very high streamline density suggesting extremely efficient fluid transport. Finally, when *v_L_ > v_U_*, streamlines suggest a passage from lower to upper chambers; specifically, random scaffolds exhibit more dispersed flow with reduced density reflecting decreased velocity in the upper chamber and less intense flow. In contrast, for aligned scaffolds with fibers perpendicular to flow direction, streamlines are more concentrated towards the lower chamber but show reduced density; while for aligned scaffolds with fibers directed as flow, direction remains well directed albeit at lower streamline density.

### 2.4 Microfluidic Experiments

**Figure 10:**
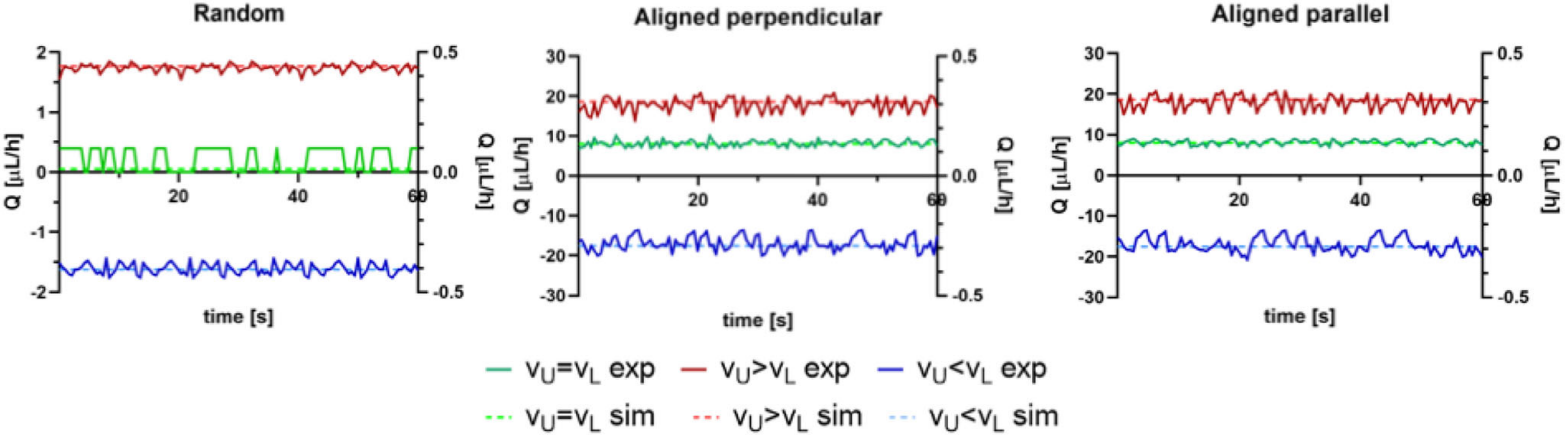
Experimental (exp) and simulated (sim) flow rates across the scaffold (Q_L –_ Q_U_) under three different flow conditions (color‐coded). The solid lines represent experimental data, while the dashed lines indicate simulation results. The secondary y‐axis is used for v_U_ = v_L_ because its values are an order of magnitude smaller, making them otherwise unreadable on the primary axis.

The results of experimental pressure measurements and CFD simulations were compared to evaluate the accuracy of the simulations in reproducing flow behavior in the microfluidic device. Experimental flow rate data were obtained through direct measurements inside the OoC chambers for both fiber arrangements (random and aligned) and for the three flow configurations (equal velocities or with a higher velocity in one chamber). The graphs in **Figure** **10** illustrate experimentally measured flow rates (Q) across the scaffold (expressed as *Q = Q_L_ – Q_U_*) for different scaffold morphologies (random and aligned) and flow configurations. The flow rate *Q = Q_L_ – Q_U_* shows whether the flow rate at the outlet of the upper chamber (*Q_U_*) is greater than that in the lower chamber (*Q_L_*), revealing the direction of flow across the membrane. In the OoC containing the R‐PLA scaffold (Figure 10A), the flow rate across the scaffold exhibited lower values of Q than those observed in the other configurations (Figure 10B,C). This suggests that the disordered fiber arrangement creates a more turbulent flow, increasing resistance to fluid passage, consistent with the experimentally evaluated lower permeability (*K*).

Both the experimental and simulation results showed that when *v_U_ > v_L_*, the flow rate at the outlet of the upper chamber was lower than that of the lower chamber, suggesting a net flow from the upper to the lower chamber through the scaffold. A similar trend was observed for *v_U_ = v_L_*, although the difference in flow rates (*Q_L_ – Q_U_*) was nearly zero, indicating minimal net flow across the scaffold. Conversely, when *v_U_ < v_L_*, the opposite behavior occurred, with flow preferentially moving from the lower to the upper chamber. For the random scaffold under the *v_U_ = v_L_* condition, the measured flow rates were within the sensitivity range of the flow sensor, meaning that the precision of the recorded values is limited, and small fluctuations may be attributed to sensor resolution rather than actual flow variations. In general, the flow rate fluctuations observed in the experimental data reflect dynamic variations in the system, likely due to microfluidic instability or sensor sensitivity. Overall, the agreement between experimental and simulated trends supports the reliability of the computational model in predicting flow behavior.

## 3 Discussion

Dual‐chamber microfluidic systems with an integrated porous scaffold between the two compartments have recently been utilized for dynamic cell co‐culture models to mimic fibrous tissues.^[^ ^29^, ^30^, ^31^ ^]^ When fabricated by electrospinning as support for cells, scaffolds can exhibit different morphologies in fiber distribution, porosity, and thickness.^[^ ^32^ ^]^ Furthermore, the 3D structure of our scaffold mimics the natural ECM, providing a supportive environment for cell growth while modulating barrier effects. This three‐dimensionality arises from the scaffold‐controlled thickness (35–70 microns), sufficient to form an interconnected fibrous network rather than a thin, 2D membrane. Unlike ultrathin membranes, which primarily allow cell adhesion on a flat surface, electrospun scaffold architectures enable a biomimetic environment for cell adhesion and growth.^[^ ^33^, ^34^ ^]^ In fact, electrospun scaffolds offer superior structural complexity and porosity control if compared to other porous systems, and they allow for precise modulation of paracrine signaling by modifying their architecture and thickness through different processing parameters.^[^ ^32^ ^]^

The influence of fiber orientation on the porosity of scaffolds has been investigated in various studies. Our findings show that random scaffolds exhibit a porosity of 75% compared to 64% for aligned scaffolds, which aligns with existing literature that explores the relationship between scaffold configuration and porosity and is related to inherent structural differences resulting from the electrospinning process.^[^ ^28^ ^]^ The scaffold composed of random fiber exhibits a less ordered fiber arrangement than aligned ones, resulting in higher overall porosity. In contrast, the aligned fibers pack more closely, reducing the void volume in the structure and resulting in lower porosity.^[^ ^35^, ^36^, ^37^, ^38^ ^]^

SEM images corroborate this hypothesis by showing clear differences between electrospun fibers with aligned and randomized (R‐PLA) orientations (Figure 1A). In particular, the denser fiber network formed by aligned fibers (Figure 1A) compared to randomly oriented ones (Figure 1B) is consistent with the different porosity evaluated between the two systems.

From a fiber diameter perspective (Figure S1A, Supporting Information), both configurations show a similar distribution, slightly narrower for A‐PLA samples, with a peak of ≈1 µm. The slight difference in mean fiber diameter (1.12 µm for R‐PLA and 1.02 µm for A‐PLA) has already been reported in the scientific literature and is reasonably attributed to the mechanical drawing force generated by the high rotational velocity of the collector, which leads to fiber elongation and jet stretching.^[^ ^25^ ^]^The efficacy of the orientation of the fibers was quantitatively assessed by orientation analysis (Figure S1B, Supporting Information), where aligned fibers show a sharp peak ≈0°, indicating a predominantly parallel arrangement, while randomized fibers show a wider and uneven distribution, without a preferential direction. The implications of these differences are significant for applications in tissue engineering. Fiber orientation is known to influence cellular response: aligned fibers can promote the elongation and orientation of cells in specific directions, a critical aspect for the regeneration of anisotropic tissues such as muscles, tendons, and nerves. Conversely, randomized fibers may be more suitable for applications where homogeneous cell growth in all directions is preferable, such as in tissues with a less organized extracellular matrix.

Scaffold properties influence permeability and fluid characteristics in dynamic environments.^[^ ^39^, ^40^ ^]^ However, water permeability is rarely measured systematically in scaffolds, with vapor permeability being the most commonly assessed parameter.^[^ ^17^, ^41^, ^42^ ^]^ In this study, a device was designed to quantitatively measure the water permeability of scaffolds following ISO 7198:2016, thereby improving the reliability of simulation results concerning flow characteristics and scaffold properties.

This work analyzes the relationship between scaffold permeability and fiber alignment on flow characteristics within a microfluidic device. CFD simulations at macroscopic and microscopic scales, supported by experimental comparisons, were used to examine various flow configurations. The goal is to evaluate how fiber alignments affect scaffold permeability and its ability to sustain liquid flows in OoC applications, with a focus on regulating shear stresses on scaffold surfaces.

In particular, the OoC was studied under two conditions: equal initial flow rates in both chambers (42 µL h^−1^) and a ten‐fold higher flow rate in one chamber (420 µL h^−1^)). The minimum flow rate of 42 µL h^−1^ (equivalent to 1 mL day^−1^) was chosen as it theoretically allows adequate oxygenation to cell cultures. More in detail, according to Trenton et al.,^[^ ^43^ ^]^ under standard culture conditions (100% humidity, 5% CO_2_, 37 °C), the dissolved oxygen concentration in a culture medium can be estimated to be 0.194 mM.^[^ ^44^ ^]^ Assuming for cells an O_2_ consumption of 0.8 nM/10^6^ cells/s,^[^ ^44^ ^]^ this flow rate can be adopted for cultures up to ∼ 10^7^ cells, despite the low oxygen permeability of polymethyl methacrylatep (PMMA).^[^ ^45^ ^]^

On the other hand, the highest flow rate exhibited a maximum shear stress trend of 10^−3^ Pa (0.01 dyn cm^−^^2^) along scaffold fibers. This value is lower than the maximum shear stress tolerable by various types of epithelial cells, which varies depending on cell type and experimental conditions. For instance, one study observed that Caco‐2 cells show changes in their characteristics when exposed to shear stress between 0.01 and 0.03 dyn cm^−^^2^.^[^ ^46^ ^]^ In addition, another study developed reusable culture chambers to apply gastrointestinal shear stress in the range of 0.002–0.08 dyn cm^−^^2^ to HT29‐MTX epithelial cells, observing a rearrangement into 3D villi‐like structures and a significant increase in the thickness of the mucus layer under flow compared to static conditions.^[^ ^47^ ^]^ These studies confirm that shear stress in the order of 0.01–0.03 dyn cm^−^^2^ can affect epithelial cell morphology and function, justifying the choice of a flow rate of 1 mL day^−1^ (42 µL h^−1^) in our model. This value allows the application of flow conditions compatible with cell survival and behavior, reproducing the mechanical environment typical of epithelial tissues in vitro.

The role of fluid dynamics, pressure, and shear flow profiles in biological functions has been extensively studied regarding cellular regulation.^[^ ^48^, ^49^ ^]^ Although the effects of fiber alignment and scaffold permeability on flow characteristics remain unclear, existing evidence provides some insights into their correlation. A study by Cui et al. demonstrated the fabrication and characterization of biocompatible Chitosan/Poly (ε‐caprolactone) composites via electrospinning techniques, yielding both aligned and random nanofibers.^[^ ^50^ ^]^ The results indicated that aligned nanofibers had a lower average diameter and porosity than random ones. In addition, the mechanical properties of aligned nanofibrous scaffolds were superior to those that were randomly oriented. This suggests that the permeability of aligned nanofibers may be lower than that of random nanofibers due to their smaller mean diameter and lower porosity; permeability is inversely proportional to pore size and proportional to porosity.

Fasolino et al. developed a process that combines electrospinning, spray coating, and solid‐state polymerization to create scaffolds with aligned and random fibers.^[^ ^51^ ^]^ For biological analysis, they used a neuroblastoma‐derived human cell line (SH‐SY5Y) to assess cell maturation on random and aligned eumelanin microfibers through confocal analyses and specific markers for differentiating neurons (expression of βIII tubulin and GAP‐43). Their results showed that the random arrangement of microfibers provides a more homogeneous environment than aligned fibers, promoting better cellular responses regarding cell proliferation and morphology. Hence, it could be hypothesized that the random arrangement of microfibers positively affects scaffold permeability by providing a more uniform environment conducive to improved cell responses related to proliferation and morphology.

Chen et al. employed numerical simulations and theoretical modeling to examine how length, channel height, and scaffold permeability affect flow in a microfluidic system.^[^ ^22^ ^]^ These factors influenced flow amount, penetration rate, and shear stress on the channel walls; increased scaffold permeability reduced flow and penetration rates in the upper channel while increasing them in the lower channel. However, the impacts of microscopic fiber distribution and morphology – and their correlation with scaffold permeability on flow characteristics in a dual‐flow regime with two inlet velocities – remain unknown.

The CFD method is commonly used to study fluid characteristics inside dynamic systems without evaluating elastic deformations.^[^ ^52^, ^53^, ^54^ ^]^ In this work, simulations were implemented inside the microfluidic device at both macroscopic and microscopic scales. At the macroscopic scale, the scaffold was simplified as a porous and permeable solid At the microscopic scale, real geometries of fibers obtained from SEM images were used to give morphological features to the top and bottom surface of the porous solid, reducing the computational cost and allowing the incorporation of measured porosity and permeability of scaffolds with aligned and random fibers into the microscale simulations. The arrangement of aligned fibers relative to flow direction (parallel or perpendicular) was also evaluated. This strategy has been employed in similar studies,^[^ ^55^, ^56^ ^]^ but to the best of our knowledge, it was never adopted for microfluidic systems integrating electrospun scaffolds.

The results from both simulations elucidated the dependencies between scaffold flow rate and permeability concerning shear stress and pressure differences between chamber inlets and outlets. They also indicated that variations in internal scaffold structure based on fiber orientation directly influence measured porosity. These differences are relevant for specific applications where scaffold porosity affects barrier or permeation properties in tissue engineering. Analysis of pressure drop profiles between random and aligned scaffolds revealed interesting variations reflecting different scaffold structures’ impact on microfluidic flow.

Specifically, streamline results from Figure 8 indicated that the chip containing the A‐PLA scaffold with fiber parallel to flow exhibited higher fluid transport efficiency across the scaffold, especially when *v_U_ > v_L_*, highlighting the crucial role of fiber orientation in optimizing flow control. In contrast, random scaffolds and those aligned perpendicular to flow offered less directionality and greater flow dispersion under certain conditions.

These observations are essential for designing scaffolds intended for operation under varying flow conditions, allowing tailored design for specific applications. A‐PLA scaffolds demonstrated greater uniformity in pressure drop profiles potentially promoting more homogeneous flow distribution across the scaffold and reducing turbulence.^[^ ^57^ ^]^

Analysis of different flow configurations’ impact on pressure drop profiles highlighted variations in microfluidic flow behavior. When *v_U_ = v_L_* (Figure 3), the system tends toward balance with relatively uniform velocity, pressure, and shear stress distributions, suggesting a controlled, less turbulent flow ideal for applications requiring uniform diffusion across scaffolds. Conversely, when *v_U_ =* 10 × *v_L_* (Figure 4), more asymmetry and instability are observed, leading to enhanced mixing and turbulence beneficial for applications requiring vigorous dispersion. In summary, selecting appropriate flow conditions is crucial for tuning fluid transport across chambers in multicompartmental microfluidic systems, impacting flow behavior, pressure distribution, and shear stress.

Moreover, the adaptability of our simulation framework is also achievable by defining membrane thickness, porosity, and permeability as key parameters that enable the evaluation of fluid transport across various porous structures, including track‐etched membranes with tunable cylindrical pore arrays. Integrating direct permeability measurements (ISO 7198:2016) and pore size distribution enhances model validation, improving flow rate and shear stress predictions. These refinements also allow for direct CFD simulations of flow through individual pores, increasing the accuracy of permeability estimations.

An interesting finding was that both simulations and the experimental evidence indicated fluid flow from the upper to lower chamber when input velocities were equal for both aligned and random fibers. This phenomenon likely relates to transmembrane pressure differences creating necessary pressure gradients to drive liquid flow across the scaffold from one side to another.^[^ ^22^, ^58^ ^]^

Comparison of experimental results with simulated outcomes showed good alignment, confirming the reliability of the computational model. This consistency underscores the model's robustness and validates its applicability for accurately simulating fluid dynamics in similar experimental conditions. The analysis of flow rate distributions helped to characterize scaffold permeability under different flow conditions, revealing preferential flow directions based on velocity ratios.^[^ ^59^, ^60^ ^]^ Identifying these patterns is crucial for optimizing microfluidic system design, as it allows for better control over flow distribution, minimizes unwanted disturbances, and ensures reproducibility. Additionally, for conditions where flow rates were within the sensitivity range of the sensors, measurement precision becomes a limiting factor, highlighting the need for refined experimental setups to improve data accuracy.

This work elucidated how fiber alignment regulates flux and shear stresses experienced by cells seeded onto scaffolds—providing a foundation for evaluating flux characteristics in similar chips based on scaffold permeability—and promoting designs for tissue‐specific flux chips suitable for developing dynamic microfluidic models. However, some limitations need to be recognized. In particular, the evaluation of permeability was mainly based on water flow measurements, without including macromolecule transport experiments. More in‐depth permeability studies, for example through the use of tracer molecules such as FITC‐labeled Dextran, would be critical for a more complete characterization of scaffold barrier properties, especially under physiological conditions where diffusion depends on molecular size. Future integration of these analyses may provide a more accurate understanding of scaffold behavior in dynamic cell co‐culture models or organ‐on‐chip applications. In addition, the limitation of experimental sensitivity in detecting very low fluxes highlights the need to further optimize the measurement system to obtain more accurate quantitative data, particularly for fluxes comparable to physiological fluxes.

Overall, this research demonstrated how sensitive electrospun scaffold morphology is regarding fluid flow while establishing quantitative approaches for describing correlations between flow fields and scaffold properties.

## 4 Conclusion

In conclusion, this study provides a comprehensive understanding of the influence of fiber orientation in electrospun scaffolds on permeability properties and flow dynamics in microfluidic devices. By adopting an electrospun PLA scaffold composed of random or aligned fibers, we demonstrated that fiber configurations significantly impact flow characteristics, including velocity and pressure distribution within the microfluidic device. Specifically, scaffolds with aligned fibers exhibited greater uniformity in pressure drop profiles and enhanced fluid transport efficiency, particularly when the flow velocity in the upper chamber exceeded that in the lower chamber. Conversely, scaffolds with randomly arranged fibers displayed greater flow dispersion, accompanied by increased variability in pressure and shear stress values.

A notable outcome of this research was the successful correlation between experimental data with CFD simulations, providing robust validation of the models employed. The simulations at the microscale further allowed visualization at the level of individual fibers, highlighting how fiber orientation leveraged to modulate the fluid microenvironment in microfluidic applications such as OoC systems.

These findings are particularly relevant for the design of OoC devices where precise control of flow is critical, such as in dynamic cell cultures or in vitro tissue models. This study not only underscores the pivotal role of fiber orientation but also establishes a solid methodological foundation for designing scaffolds suitable for microfluidic applications. This approach has the potential to inspire innovative strategies in developing advanced in vitro models, significantly advancing the field of biomedical technology.

## 5 Experimental Section

### Materials

PLA (polylactic acid) type 2002D, supplied by NatureWorks (Minneapolis, MN, USA), was used to fabricate the scaffolds. For the homogeneous solution of the PLA before the electrospinning process, acetone (Ac) and chloroform (TCM), purchased from Sigma‐Aldrich (St. Louis, MO, USA), were used as solvents. To print the device for measuring permeability, commercial Clear v4 resin was used, supplied by Formlabs (Somerville, MA, USA). The resin was employed in a stereolithographic 3D printer, ensuring a smooth and uniform surface for driving liquid flows.

For the fabrication of the dual‐chamber microfluidic device, PMMA (polymethyl methacrylate) was used as the structural material, supplied by Clarex, Nitto Jushi Kogyo Co. Ltd. (Osaka, Japan), with ethanol as the solvent for processing PMMA. A connection system was employed to connect the OoC to the syringes, which included mini male luer plugs and fluid connectors, silicone and polytetrafluoroethylene (PTFE) tubing, ethyltrifluoroethylene (ETFE) ferrules, and perfluoroalkoxy (PFA fittings, all purchased from Microfluidic ChipShop (Jena, Germany). All reagents and solvents used were ACS grade (purity >99%), ensuring purity and reproducibility of measurements.

### Electrospinning of the Scaffold with Random and Aligned Fibers

For scaffold fabrication, PLA was dissolved at a concentration of 10 wt% in a solvent mixture of TCM and Ac (2:1 vol) at room temperature, with continuous magnetic stirring overnight to achieve a homogeneous solution. A semi‐industrial electrospinning machine (NF‐103, MECC CO., LTD., Japan) equipped with a grounded cylindrical rotating collector (diameter = 10 cm) was used to fabricate the scaffolds. The polymer solution was loaded into a 5‐mL syringe fitted with a 19‐gauge stainless steel needle. The following constant parameters were established: flow rate of 0.7 mL hr^−1^, needle‐to‐collector distance of 17 cm, high voltage of 17 kV, temperature of 25 °C, and processing time of 180 min (Table S1, Supporting Information). After the electrospinning process, the scaffolds were dried for 48 h under a fume hood to remove any residual solvents.

Two types of scaffolds were produced: one with randomly arranged fibers and the other with aligned fibers. The fiber orientation within the electrospun scaffolds was precisely controlled by adjusting the angular velocity of the rotating collector during the electrospinning process. For random fiber orientation, a low angular velocity (10 rpm) was employed while for aligned fiber orientation, a high angular velocity (3000 rpm) was used.

Given that PLA is known for its hydrophobic characteristics, the scaffolds underwent air‐cold plasma treatment using a Plasma Cleaner (AP300 System, NORDSON, Westlake, OH, USA) with a radio frequency of 13.56 MHz and power of 50 W. The chamber was depressurized to 0.2 mbar and stabilized for 5 s before air was introduced to trigger the plasma reaction. Ventilation was conducted at room pressure for 60 s, and this treatment was applied to both sides of each scaffold for 60 s each. The random scaffolds had an approximate thickness of 70 µm, while the aligned scaffolds measured ≈35 µm in thickness.

### Porosity Measurement

The porosity (φ) of the PLA scaffold scaffolds was calculated according to Equation (1)^[^ ^27^ ^]^:

(1) $$\varphi =1-\frac{\rho_{scaffold}}{\rho_{PLA}}$$

where 𝜌_𝑠𝑐𝑎𝑓𝑓𝑜𝑙𝑑_ is the apparent density of the scaffold and 𝜌_PLA_ is the density of the polymer matrix used in this work 𝜌_𝑠𝑐𝑎𝑓𝑓𝑜𝑙𝑑_, obtained from the technical datasheet obtained from the supplier. The resulting value (φ) represents the porosity of the PLA scaffold scaffolds as a dimensionless fraction. To express this value as a percentage, it was multiplied by 100.

For this measurement, PLA scaffolds (N = 5) were precisely laser‐cut into circular shapes with a specific diameter, and their mass (*M_scaffold_)* was measured using a precision balance. The scaffolds' volumes (*V_scaffold_*) were determined based on known geometrical formulas. The density (*ρ_scaffold_*) of the PLA scaffolds was calculated according to Equation (2):

(2) $$\rho_{scaffold}=\frac{M_{scaffold}}{V_{scaffold}}$$

### SEM Characterization

The morphology of the scaffolds was characterized using scanning electron microscopy (SEM). For the SEM analysis, circular scaffolds with a diameter of 10 mm were cut and attached to aluminum mounts using carbon tape. To enhance electrical conductivity for SEM examination, a gold coating was applied to the scaffolds using sputter deposition for 60 s under argon (Sputtering Scancoat Six, Edwards). SEM Images were obtained using a FEG‐ESEM QUANTA 200 (FEI Company, Hillsboro, USA) at an accelerated voltage of 10 kV. This configuration allowed for the acquisition of high‐resolution images of the scaffold surface, facilitating the study of fiber distribution and orientation.

Fiber diameter and orientation were analyzed from SEM images with two dedicated plugins for ImageJ, i.e., DiameterJ and OrientationJ (v2.0.5), respectively. These measurements were then used to determine the distribution of fiber diameter and orientation (N = 30).

### Design of a Custom Permeability Measuring Device

**Figure 11:**
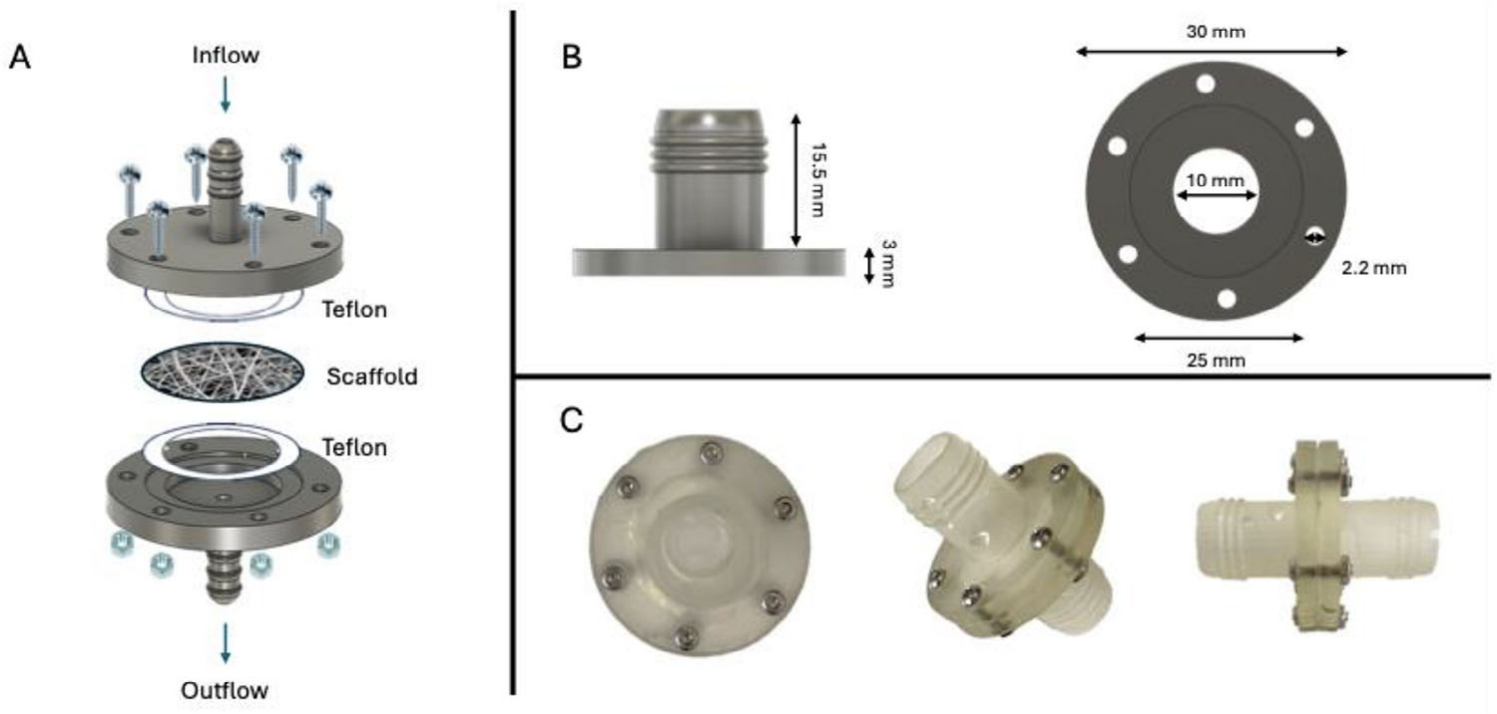
CAD and images of the system designed for permeability measurement: A) Disassembled view of the system, illustrating the fluid inflow and outflow, along with Teflon components and a porous scaffold. B) Dimensions of the main component, with side and top views. C) Photographs of the assembled device, showing top, isometric, and side views.

To assess the permeability of the scaffold, in this work, a customized device was designed, with **Figure** **11** detailing its components and configuration. In Figure 11A, the disassembled view is shown, including the fluid inflow and outflow pathways, two Teflon components, and the porous scaffold. In Figure 11B, geometric dimensions in millimeters are shown. Moreover, in Figure 11B, (right panel), two concentric circles are illustrated: a 10 mm, diameter opening for fluid passage and a 20 mm diameter recess housing a 1 mm‐thick Teflon spacer. This additional Teflon layer ensures that the scaffold rests without stress against the molded material and prevents liquid bypass over the scaffold by sealing the system. Figure 11C displays photographs of the assembled device in top, isometric, and side views.

A circular opening with a diameter of 1 cm (corresponding to an area of 0.78 cm^2^) was selected for scaffold insertion. The CAD design included a constant opening, holes for screws to seal the structure, and a cove for housing a 1 mm thick Teflon ring used to create a tight seal between the scaffold and the device housing, thus preventing leakage around the scaffold edges. The two components shown in Figure 2B were fabricated through a stereolithographic (SLA) 3D printer (Formlabs Form2, Somerville, MA, USA) and the transparent Clear V4 resin. After printing, the components underwent two washes in isopropanol, each lasting 15 min, to remove any residual non‐solidified resin and ensure a clean surface free from impurities. The components were then allowed to dry under a chemical fume hood to eliminate any remaining solvents or vapors.

Finally, the components were subjected to a curing process in a Form Cure tank (Formlabs, Somerville, MA, USA) for 30 min at 60 °C. This step ensures the complete solidification of the resin, thereby enhancing the strength and durability of the device.

A detailed schematic diagram of the complete experimental setup for permeability measurements, including labeled inflow and outflow ports and scaffold placement within the device, is provided in Figure S2 (Supporting Information).

### ISO Permeability Measurement for Aligned and Random Scaffolds

To use the printed device in conjunction with the electrospun scaffold, the two printed parts and the scaffold were assembled. Before assembly, the thickness of the scaffold was measured using a thickness gauge. A layer of Teflon was then attached to the designated space, and the scaffold was carefully laid down to avoid any creases. The two printed parts were aligned and secured with nuts and screws to create a sealed assembly, preventing leakage during liquid flow.

Aligned and random electrospun scaffolds (N = 4 per group) were tested for permeability following ISO standards. To generate a pressure of 16 kPa as the driving force for water flow, hydrostatic pressure was applied using a system positioned at a height of 1.63 m above the scaffold, connected with a tube with a diameter of 1 cm. The device containing the scaffold was immersed in water until fully soaked, after which it connected to the water delivery system.

Once a steady‐state flow was established, a stopwatch was started. After 1 min, the mass of the collected water that had passed through the scaffold was weighed. This mass corresponds to a volume of water and can be used to calculate the volumetric flow rate over time. According to Darcy's law (Equation 3), this flow rate is related to the permeability (*k*) of the material by its geometric characteristics:

(3) $$Q=-kA\frac{\Delta P}{\mu L}$$

In this equation, A represents the cross‐sectional area through which flow occurs (m^2^), L is the length of the flow path (m), ΔP is the pressure difference (Pa), and µ is the fluid viscosity (Pa s).

### Chip Design and Fabrication

The microfluidic device was designed using Autodesk Fusion 360 CAD as a multilayer structure, consisting of seven stacked layers with varying thicknesses (0.5 and 2 mm) (Figure 2A). The fabrication of the device was performed using a CO₂ laser cutter (Maitech Advanced Machinery, 40 W, Varese, Italy) controlled via AutoLaser software. PMMA sheets of 0.5 and 2 mm thickness were selected for manufacturing, ensuring compatibility with the desired microfluidic architecture. The cutting mode, power, and speed settings for the laser were adjusted according to the requirements of each layer. Specifically, for PMMA sheets of 0.5 mm thickness, the laser was set to a power of 15% and a speed of 15 mm s^−1^. For 2 mm sheets, the power was increased to 30% while maintaining the same speed of 15 mm s^−1^, and the cutting process was repeated twice to ensure complete cutting. Engraving operations were performed at a power of 15% and a speed of 300 mm s^−1^.

The layers and scaffold were assembled using a multilayer assembly technique with a laboratory hot press consisting of two heated plates. Before assembly, the layers were cleaned of any polymer residue from the laser cutting process by placing them in an ultrasonic bath with 70% ethanol for 2 min. The layers were then pressed with ethanol at 70 °C under 1200 psi for 180 s to achieve a strong and uniform seal.

At the end of the process, the OoC was removed from the press and flushed with air into the upper (*U*) and lower (*L*) channels using a syringe without a needle to eliminate any excess ethanol.

### Computational Simulation

The fluid dynamics of the microfluidic device were simulated using the COMSOL Multiphysics, specifically employing the porous media flow module to model single‐phase flow in pores based on Darcy's law and Brinkman's equations. These equations account for fluid movement in porous media while incorporating an additional viscous term beyond Darcy's law. In this study, the term “macroscopic” refers to the overall dimensions of the microfluidic device and scaffold (on the order of millimeters), while “microscopic” refers to the dimensions of the individual fibers and pore spaces within the scaffold (on the order of micrometers). The microscopic scale was not explicitly simulated, but the fiber diameter and membrane distance were 1 and 100 microns.

In the macroscopic configuration, to represent the scaffold between the chambers, a uniform porous solid was introduced. The chip OoC was tested under two conditions: with equal initial flow rates in both chambers (42 µL h^−1^) and with one chamber experiencing a flow rate ten times higher than the other (420 µL h^−1^). A flow rate of 42 µL h^−1^ (equivalent to 1 mL day^−1^) was chosen as performed calculations showed that ≈10^7^ cells could be oxygenated, considering the chamber geometry. Moreover, in the case with equal initial flow rates in both chambers, an equal flow rate across the surface of the membrane was intended to be applied. The second condition involves imposing a flow rate ten times higher in one chamber to investigate whether this differential flow induces a more substantial passage of fluid through the membrane. Such fluid transport could be beneficial for applications requiring enhanced mass exchange, such as modeling pathological conditions or optimizing drug delivery across the membrane.

The scaffold parameters, such as porosity and permeability, were experimentally determined, while density values were sourced from existing literature.^[^ ^60^, ^61^ ^]^ The results were analyzed in terms of velocity, pressure, and shear stress on the scaffold. Additionally, fluid flow lines were visualized under various configurations, including:

1. Uniform Distribution: Streamlines are evenly spaced regardless of flow velocity, providing a general overview of streamline arrangement.
2. Across membrane: Streamlines are generated only on the scaffold surfaces, enabling detailed analysis of flow behavior across the scaffold.

For microscopic simulations, SEM images of PLA scaffolds produced through electrospinning—both in random and aligned fiber configurations—were processed to capture the microscopic arrangement of fibers. This facilitated the creation of a 3D fiber structure using Fusion360 software. The resulting 3D scaffold was then integrated with a uniform porous solid to form a composite structure that accurately reflects the actual thickness and permeability of the scaffold.

**Figure 12:**
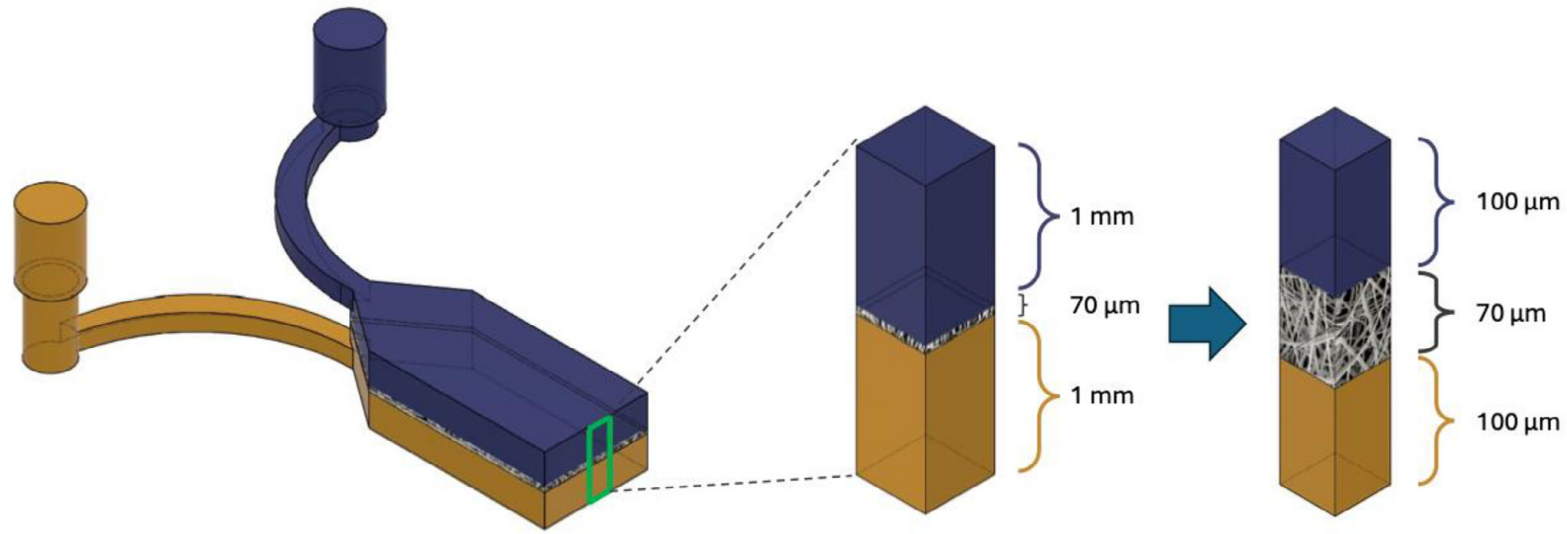
Zoomed‐in view of the fluid domain used for the simulations at the microscopic scale. The selected volume includes the electrospun PLA scaffold, characterized by its porous structure, allowing for a detailed analysis of fluid flow dynamics at the microscale.

Microscopic simulations were evaluated on a fluid volume centered on the membrane (as shown in **Figure** **12**) to assess fluid behavior through the scaffold in terms of velocity, pressure, and shear stress on fiber surfaces.

Flow lines were scrutinized to enhance understanding of fluid dynamics. In these microscopic simulations, COMSOL software was utilized, with velocity values from macroscopic simulations applied as input and output conditions in the microscopic framework. Pressure values from the macroscopic scale were similarly used as input and output conditions along the yz planes in the flow direction, while all other surfaces were treated as open boundaries. The flow inlets were located at the ends of the upper and lower channels, far upstream from the scaffold, to allow for flow development before interacting with the scaffold.

Using the Brinkman model, system equations were solved by varying inlet velocity values consistent with those from the macroscopic case. For aligned scaffolds, simulations considered both parallel and perpendicular flow directions relative to fiber alignment.

### Microfluidic Experiments

To validate the simulation results, flow velocity sensors (Sensirion SLF3S‐0600, Stäfa, Switzerland) were installed to accurately measure flow velocity and direction within the chip chambers and through the scaffold. The flow was delivered using two syringe pumps and maintained for a sufficient period to reach a steady state flow condition before measurements recording. To compare the experimental data with the simulation results, the flow rate passing through the scaffold was calculated based on the pressure difference obtained from the microscale simulation using the Darcy Law. This computed flow rate was then compared to the experimental data by subtracting the outlet flow rates from the inlet flow rates in the two chambers, using the readings from the flow sensors. The resulting data were analyzed and visualized using GraphPad Prism 8 Software.

## Conflict of Interest

The authors declare no conflict of interest.

## Author Contributions

F.L. and M.T. conceptualized the idea for the study. E.C., M.T., C.D.M, F.L., and V.L.C. designed the methodology. F.L. and M.T. developed the microfluidic device. C.D.M fabricated and characterized the scaffold. E.C. performed computational and experimental validation. E.C. prepared for writing the original draft. E.C., M.T., C.D.M, F.L., and V.L.C. wrote, reviewed, and edited the manuscript. All authors have read and agreed to the published version of the manuscript.

## Supporting information



Supporting Information

## Data Availability

The data that support the findings of this study are available from the corresponding author upon reasonable request.
