# Supplementary material for: Impact of Fiber Orientation in Electrospun PLA Scaffolds on Fluid Dynamics in a Custom Microfluidic Device
Source: Adv Healthc Mater. 2025 Jun 17;14(25):2500378. doi: 10.1002/adhm.202500378 (PMC12477572; doi:10.1002/adhm.202500378)
Supplement: Supplementary file 1 — Supporting Information [file ADHM-14-0-s001.pdf]

ADVANCED HEALTHCARE MATERIALS

Supporting Information

for *Adv. Healthcare Mater.*, DOI 10.1002/adhm.202500378

Impact of Fiber Orientation in Electrospun PLA Scaffolds on Fluid Dynamics in a Custom Microfluidic Device

Elisa Capuana, Maria Testa, Chiara Di Marco, Francesco Lopresti and Vincenzo La Carrubba*

Impact of fiber orientation in electrospun PLA scaffolds on fluid dynamics in a custom microfluidic device

Elisa Capuana[1], Maria Testa[1,2], Chiara Di Marco[1], Francesco Lopresti*[1], Vincenzo La Carrubba[1]

[1] Department of Engineering, University of Palermo, Palermo, Italy

[2] Department of Biomedicina, Neuroscienze e Diagnostica avanzata (Bind), University of Palermo, Palermo, Italy

E-mail: francesco.lopresti01@unipa.it

Keywords: electrospinning, fiber alignment, microfluidic devices, Organ-on-Chip, computational fluid dynamics (CFD), liquid permeability

Supporting Information

1. Additional Experimental Details

1.1. Electrospinning Process

To obtain both random and aligned polylactic acid (PLA) membranes, the electrospinning process was conducted under controlled conditions, as detailed in the main text. The flow rate, needle-collector distance, and applied voltage were optimized to ensure consistent fiber formation. A summary of the parameters used during the electrospinning process is provided in **Table S1**.

Table S1. Electrospinning parameters

Parameter	Value for Random Fibers	Value for Aligned Fibers
Flow rate	1 mL hr^{-1}	1 mL hr^{-1}
Needle-collector distance	13 cm	13 cm
Voltage	15 kV	15 kV
Temperature	25°C	25°C
Collector speed	10 rpm	3000 rpm

2. Supplementary Figures

2.1. Scaffold characterization: porosity and SEM

Scanning electron microscopy (SEM) images provided a detailed analysis of the morphology of the PLA scaffolds, distinguishing between aligned and random orientations. From **Figure S1A,** both

configurations have comparable diameters, with a distribution centered around 1 μm, but with a slight variation in relative frequency between the two conditions. Specifically, fiber size analysis showed that the average fiber diameter in random scaffolds is 1.12 ± 0.061 μm, whereas in aligned scaffolds, it is 1.02 ± 0.032 μm. Aligned fibers (A-PLA) show a parallel organization, with a narrower distribution of fiber orientation around 0° (**Figure S1B**, black line), while randomized fibers (R-PLA) exhibit a disordered orientation, with values distributed over a wide angular range.

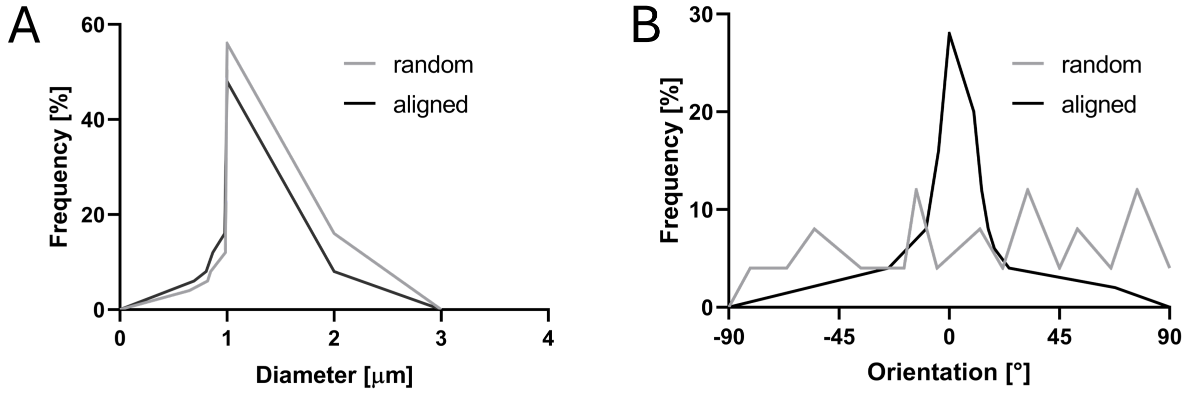

Figure S1. A) Fiber diameter size distribution for both configurations. B) Distribution of the orientation of the fibers, with angular values with respect to the principal direction.

2.2. Chip Design

The design of the microfluidic device was defined through a detailed technical drawing, which specifies the precise dimensions of each layer and structural feature (**Figure S2**). This schematic provides a comprehensive view of the arrangement of the culture chambers, microfluidic channels, and inlet and outlet ports, with measurements reported in millimeters.

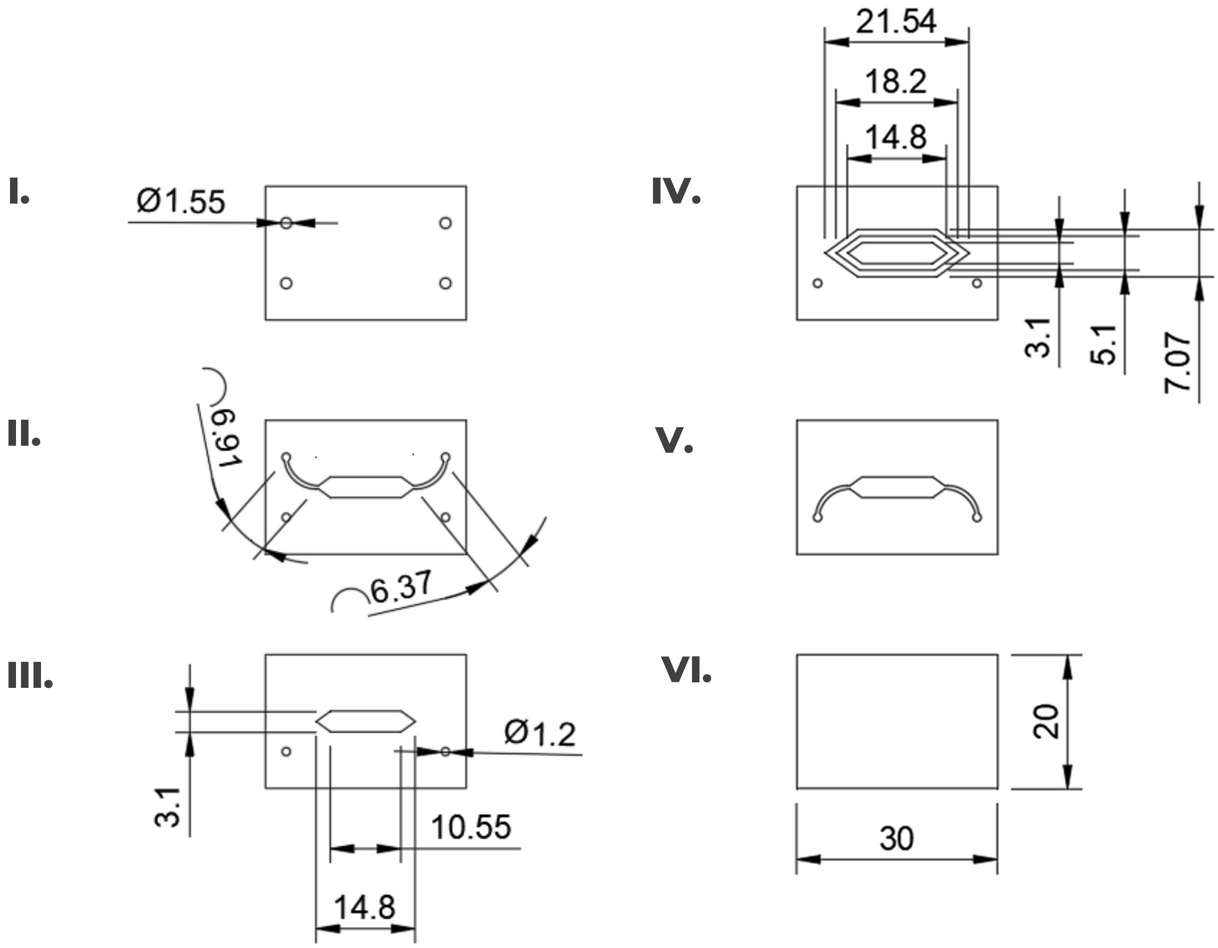

Figure S2. Detailed 2D technical drawing of the microfluidic device, illustrating the precise dimensions of each layer and structural feature. Measurements are reported in millimeters, specifying the dimensions of the culture chambers, and the positioning of microfluidic channels, inlets, and outlets**.**

2.3. Simulation results at macroscopic scale

The simulations at the macroscopic scale conducted at flow conditions $v_U < v_L$ provided insights into the velocity and pressure distributions across the membrane. These results are visualized in Supplementary **Figure S3** since they are the reciprocal results to the $v_U > v_L$ flow configuration. The same considerations provided for the latter configuration can be proposed also in this case while exchanging *U* with *L*.

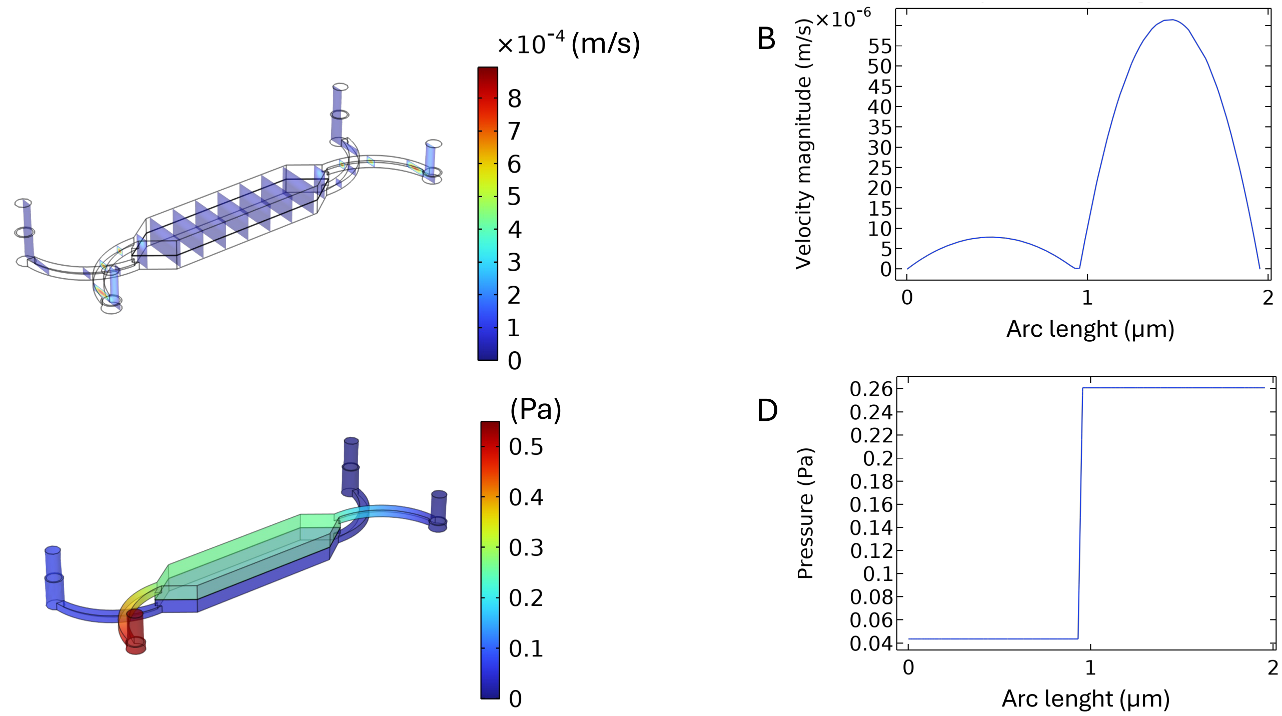

Figure S3. Simulation results at the macroscopic scale for random scaffold when $v_U < v_L$: A) velocity along five yz planes; B) velocity profile along z; C) pressure distribution; D) pressure profile along z. v_U and v_L indicate the flow velocity in the upper and lower chambers, respectively. Arc length indicates the distance along the z-axis of the device, measured in millimeters, where 0 mm represents the bottom and 2 mm the top, and along which the velocity and pressure profiles were measured.

2.4. Simulation Results at microscopic scale

The simulations at the microscopic scale under flow conditions where $v_U < v_L$ provided insights into the velocity, pressure, and shear stress distributions across the membrane during this regimen. These results are visualized in Supplementary **Figure S4** and **Figure S5**, as they are reciprocal to those obtained in the $v_U > v_L$ flow configuration. The same considerations applied to the latter configuration can also be extended to this case by simply exchanging U with L.

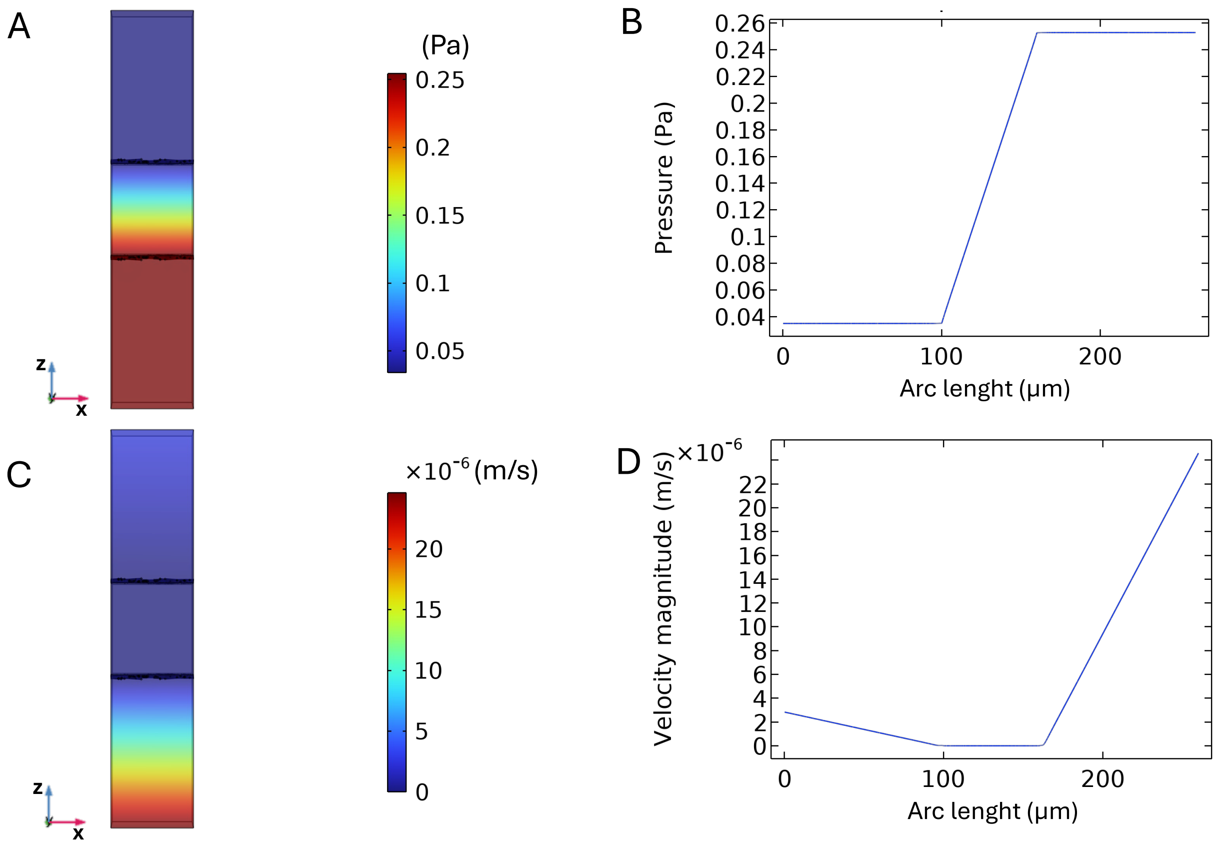

Figure S4. Simulation results at the microscopic scale for random scaffold when $v_U < v_L$: A) pressure distribution; B) pressure profile along z; C) velocity distribution; D) velocity profile along z. v_U and v_L indicate the flow velocity in the upper and lower chambers, respectively. Arc length indicates the distance along the z-axis of the domain, measured in microns, where 0 mm represents the bottom and 270 µm the top, and along which the velocity and pressure profiles were measured.

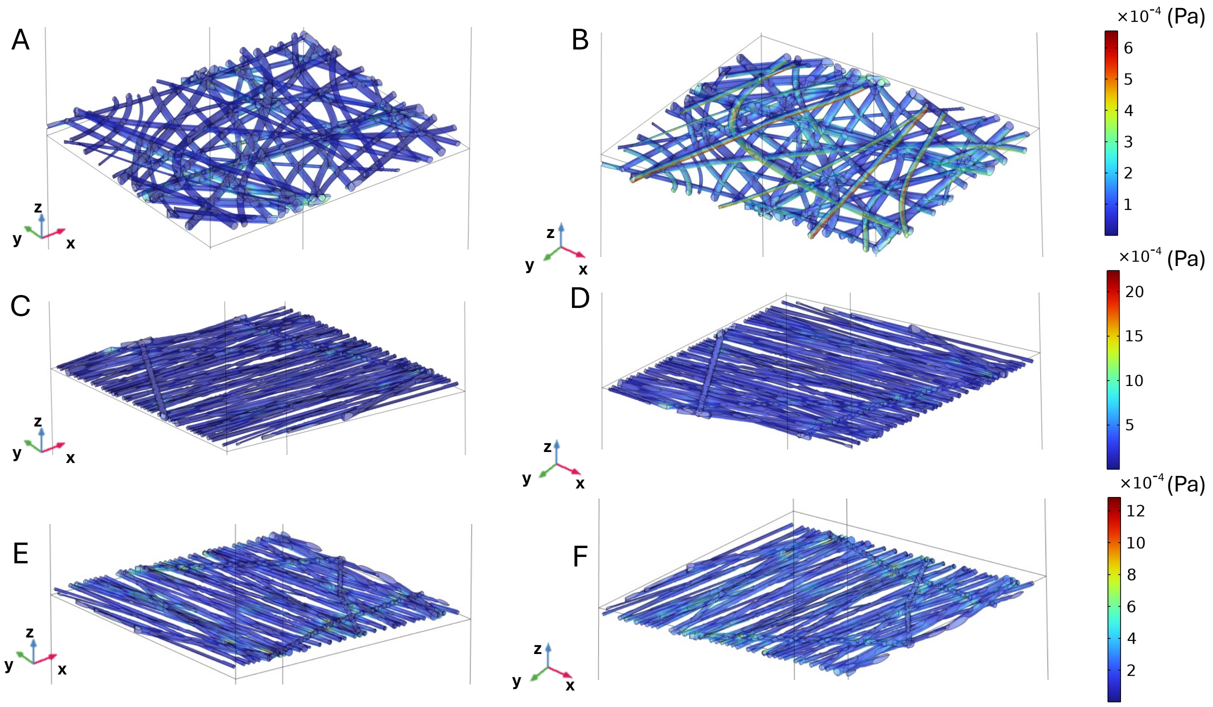

Figure S5. Shear stress on the fibers under conditions where v_L = *10* × v_U A) Upper surface of the random membrane; B) Lower surface of the random membrane; C) Upper surface of the aligned membrane with fibers oriented in the direction of flow; D) Lower surface of the aligned membrane with fibers oriented in the direction of flow; E) Upper surface of the aligned membrane with fibers oriented perpendicular to the direction of flow; F) Lower surface of the aligned membrane with fibers oriented perpendicular to the direction of flow.
